# Supplementary material for: A Comparative Phytochemical Investigation of the Greek Members of the Genus Helichrysum Mill., with Emphasis on the Local Endemic Helichrysum amorginum Boiss and Orph
Source: Plants (Basel). 2025 Jan 15;14(2):229. doi: 10.3390/plants14020229 (PMC11769484; doi:10.3390/plants14020229)
Supplement: Supplementary file 1 [file plants-14-00229-s001.zip › plants-3410106-supplementary.pdf]

Supplementary Material

A Comparative Phytochemical Investigation of the Greek Members of the Genus *Helichrysum* Mill., with Emphasis on the Local Endemic *Helichrysum amorginum* Boiss and Orph

Iordanis Samanidis [1], Nikos Krigas [2,3], Vassilis Athanasiadis [1], Ioannis Makrygiannis [1], Martha Mantiniotou [1] and Stavros I. Lalas [1,*]

[1] Department of Food Science and Nutrition, University of Thessaly, Terma N. Temponera Str., 43100 Karditsa, Greece; isaman@uth.gr (I.S.); vaathanasiadis@uth.gr (V.A.); ioanmakr1@uth.gr (I.M.); mmantiniotou@uth.gr (M.M.)
[2] Institute of Plant Breeding and Genetic Resources, Hellenic Agricultural Organization-Demeter (ELGO-Dimitra), 57001 Thermi-Thessaloniki, Greece; nkrigas@elgo.gr
[3] Department of Viticulture, Floriculture & Plant Protection, Institute of Olive Tree, Subtropical Crops and Viticulture, Hellenic Agricultural Organization-Demeter (ELGO-Dimitra), 71307 Heraklion, Greece
* Correspondence: slalas@uth.gr; Tel.: +30-24410-64783

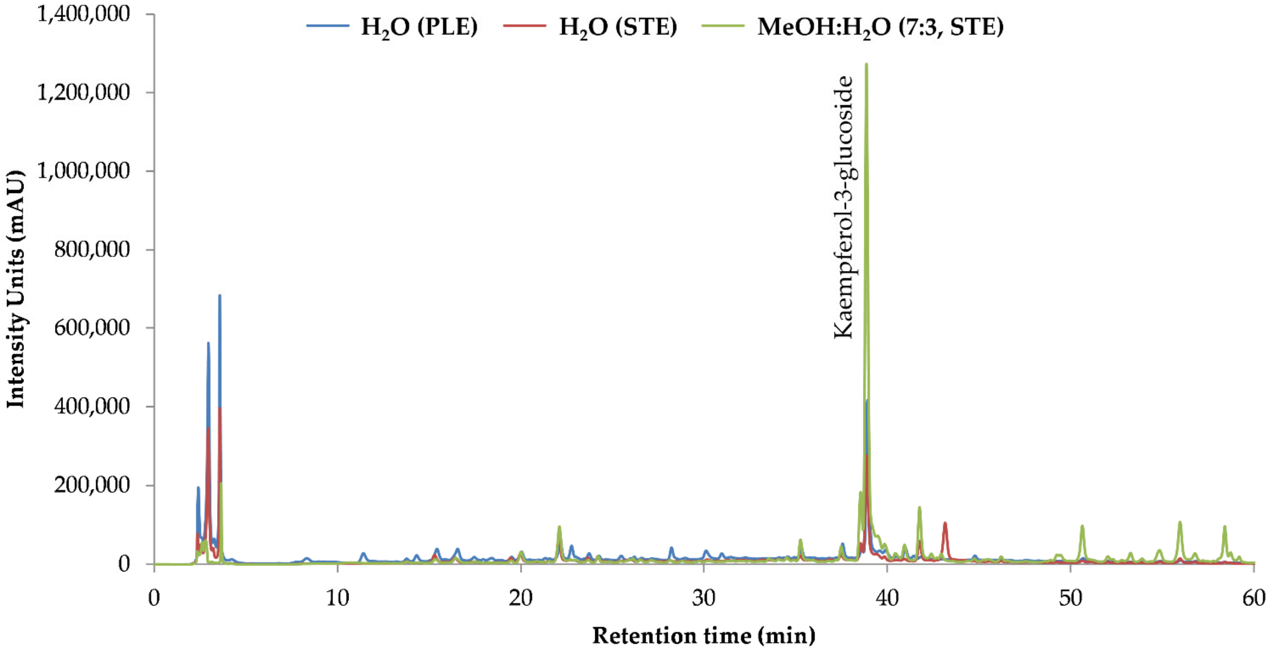

Figure S1. Chromatograms overlay of the three extracts at 266 nm.

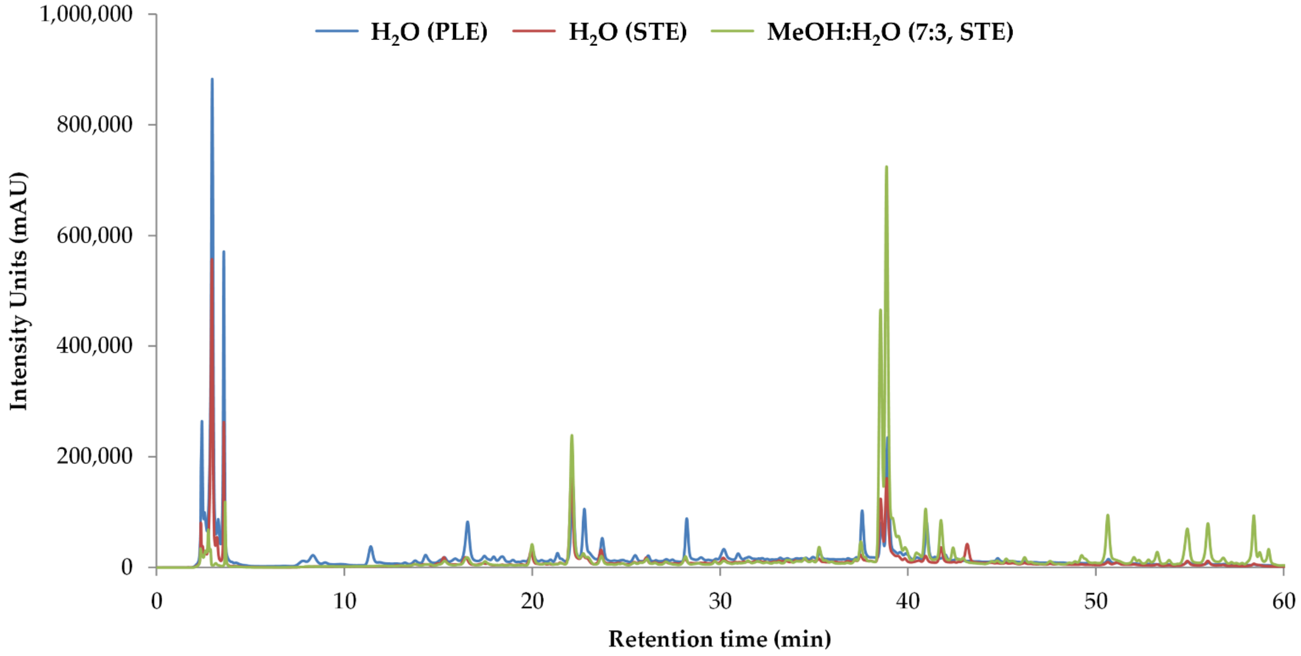

Figure S2. Chromatograms overlay of the three extracts at 286 nm.

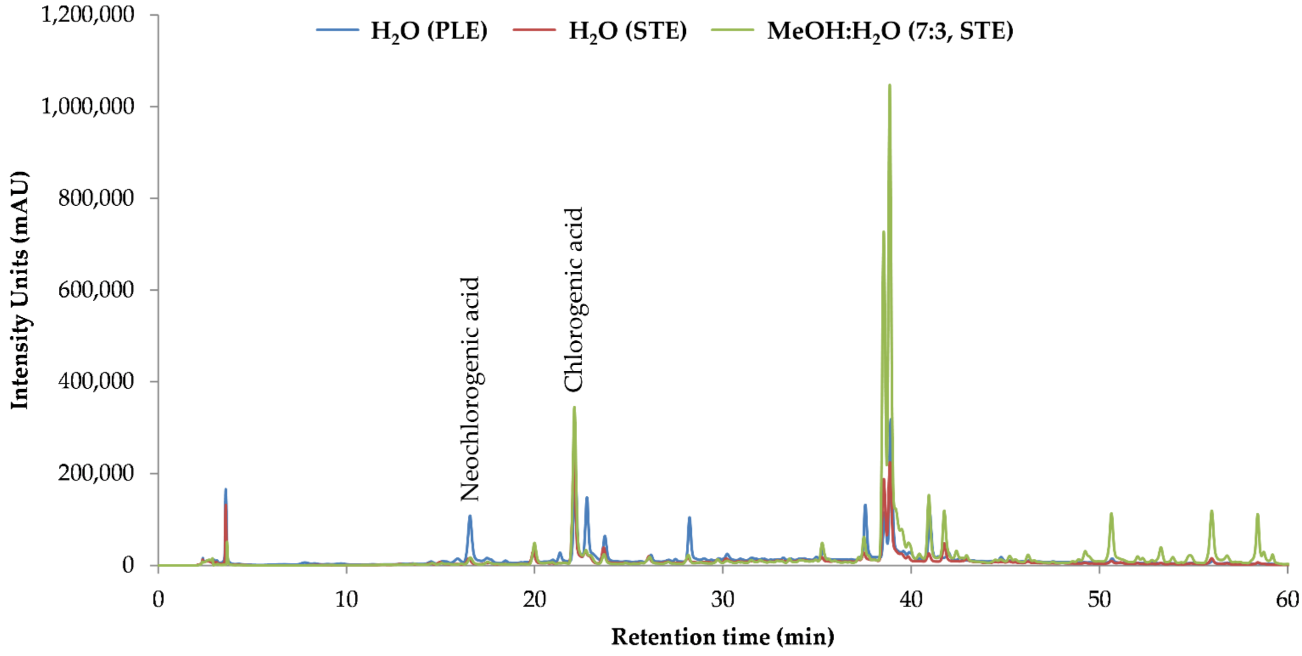

Figure S3. Chromatograms overlay of the three extracts at 321 nm.

Table S1. Native geographical distribution in the phytogeographic regions of Greece of the studied Greek endemic and the non-Greek endemic *Helichrysum* taxa.

Greek native *Helichrysum* taxa	Phytogeographical regions *	Endemic (Greece)
(HA) *H. amorginum* Boiss. and Orph. **	KiK	yes
(HT) *H. taenari* Rothm. **	Pe	yes
(HT) *H. sibthorpii* Rouy **	NE	yes
(HT) *H. doerfleri* Rech. f. **	KK	yes
(HH) *H. heldreichii* Boiss. **	KK	yes
(HO) *H. orientale* (L.) Gaertn.	Pe, WAe, EAe, KiK, KK	no
(HP) *H. plicatum* DC.	NC, NPi, SPi, StE, Pe	no
(HL) *H. luteoalbum* (L.) Rchb.	NE, NC, EC, NAe, StE, WAe, Pe, EAe, KK	no
(HSB) *H. stoechas* (L.) Moench subsp. *barrelieri* (Ten.) Nyman	IoI, StE, EC, Pe, WAe, NAe, EAe, KiK, KK	no
(HII) *H. italicum* (Roth.) G. Don subsp. *italicum*	IoI, EAe, KiK	no
(HIM) *H. italicum* subsp. *microphyllum* (Willd.) Nyman	WAe, EAe, KiK, KK	no

* According to Dimopoulos et al. [1], EAe: East Aegean Islands; EC: East Central; IoI: Ionian Islands; KiK: Kiklades; KK: Kriti and Karpathos; NAe: North Aegean Islands; NC: North Central; NE: North East; NPi: Northern Pindos; Pe: Peloponnisos; SPi: Southern Pindos; StE: Sterea Ellas; WAe: West Aegean Islands.

** For the endemic taxon's extinction risk status see reference [2].

Table S2. Annotated compounds in the methanol extract of *Helichrysum amorginum* Boiss. and Orph. (HA) based on chromatographic and spectrometric data using UPLC-HRMS/MS in negative ESI mode.

Compound	EC [M-H]$^-$	Rt (min)	RDBeq	Experimental *m/z* [M-H]$^-$	Theoretical *m/z* [M-H]$^-$	Δm (ppm)	MS/MS fragments
Gluconic acid	$C_6H_{11}O_7$	0.77	1.5	195.0512	195.0510	0.790	n.a
Quinic acid	$C_7H_{11}O_6$	0.78	2.5	191.0562	191.0561	0.464	173, 127, 111, 93, 85
Malic acid	$C_4H_5O_5$	0.82	2.5	133.0143	133.0142	0.176	115
Syringic acid 4-*O*-*β*-*D* glucopyranoside	$C_{15}H_{19}O_{10}$	2.16	6.5	359.0984	359.0972	-0.195	197, 182
Caffeoyl quinic acid 1	$C_{16}H_{17}O_9$	2.34	8.5	353.0880	353.0872	0.665	191, 179, 135
2-Isopropyl malic acid	$C_7H_{11}O_5$	2.54	2.5	175.0613	175.0601	0.647	157, 113, 85
Caffeoyl quinic acid 2	$C_{16}H_{17}O_9$	2.97	8.5	353.0881	353.0867	0.891	191, 179, 135
Caffeoyl quinic acid 3	$C_{16}H_{17}O_9$	3.07	8.5	353.0878	353.0872	-0.100	191, 179, 135
Caffeoyl quinic acid 4	$C_{16}H_{17}O_9$	3.19	8.5	353.0878	353.0872	0.155	191, 179, 173, 135
Caffeic acid	$C_9H_7O_4$	3.32	6.5	179.0.350	179.0350	0.212	135
Licoagroside B	$C_{18}H_{23}O_{12}$	3.34	7.5	431.1197	431.1189	0.582	287 $[C_{12}H_{15}O_8]^-$, 143 $[C_6H_7O_4]^-$
Caffeoyl glycerol	$C_{12}H_{13}O_6$	3.44	6.5	253.0720	253.0707	1.101	179, 161, 135
Kaempferol diglycoside	$C_{27}H_{29}O_{16}$	4.36	13.5	609.1462	609.1450	0.118	284, 271, 227, 255
Quercetin glycoside	$C_{21}H_{19}O_{12}$	4.55	12.5	463.0881	463.0877	-0.279	301, 179
Kaempferol-3-*O*-glucoside	$C_{21}H_{19}O_{11}$	5.02	12.5	447.0930	447.0927	-0.659	327, 285, 284, 283, 255, 227, 211, 181, 175
Apigenin-7-*O*-glucoside	$C_{21}H_{19}O_{10}$	5.38	12.5	431.0987	431.0978	0.812	253, 161, 117
Eriodictyol	$C_{15}H_{11}O_6$	5.40	10.5	287.0563	287.0561	0.657	259, 243, 201, 151, 125
Dicaffeoylquinic acid 1	$C_{25}H_{23}O_{12}$	5.47	14.5	515.1196	515.1190	0.137	353, 317, 299, 255, 203
Kaempferol 3-*O*-acetylhexoside 1	$C_{23}H_{21}O_{12}$	5.50	13.5	489.1040	489.1038	0.308	429, 327, 285, 284, 255, 213, 151
Kaempferol-3-*O*-malonyl-hexoside	$C_{24}H_{21}O_{14}$	5.52	14.5	533.0938	533.0931	0.134	285
Kaempferol 3-*O*-acetylhexoside	$C_{23}H_{21}O_{12}$	5.91	13.5	489.1038	489.1033	-0.060	429, 327, 285, 284, 255, 213
Kaempferol dihexoside	$C_{30}H_{25}O_{14}$	6.09	18.5	609.1248	609.1244	-0.359	463, 447, 323, 285, 243, 241, 229
Kaempferol 3-*O*-acetylhexoside 2	$C_{23}H_{21}O_{12}$	6.20	13.5	489.1040	489.1038	0.370	429, 327, 285, 284, 255, 213

Dicaffeoylquinic acid 2	$C_{25}H_{23}O_{12}$	6.36	14.5	515.1199	515.1190	0.720	353, 317, 299, 255, 203, 179, 161, 135
Quercetin	$C_{15}H_{9}O_{7}$	6.65	11.5	301.0354	301.0348	0.180	178, 151
Tiliroside	$C_{30}H_{25}O_{13}$	6.77	18.5	593.1298	593.1295	-0.512	447, 307, 285
Tribuloside	$C_{30}H_{25}O_{13}$	7.02	18.5	593.1295	593.1295	-1.018	447, 307, 285, 163
Hydroxy fatty acid	$C_{16}H_{29}O_{5}$	7.07	2.5	301.2024	301.2020	1.038	283, 171, 129, 127
Tricaffeoyl hexaric acid	$C_{33}H_{27}O_{17}$	7.21	20.5	695.1257	695.1248	0.428	533, 371, 209, 191
Kaempferol-3-*O*-sophoroside-7-*O*-glucoside	$C_{32}H_{27}O_{15}$	7.37	19.5	651.1352	651.1350	-0.589	609, 285
Kaempferol glycoside	$C_{30}H_{29}O_{14}$	7.51	16.5	613.1564	613.1552	0.149	459, 447, 297, 285, 284, 227, 211, 181
Naringenin	$C_{15}H_{11}O_{5}$	7.58	10.5	271.0610	271.0606	-0.726	271, 185, 151, 125, 119
Apigenin	$C_{15}H_{9}O_{5}$	7.64	11.5	269.0449	269.0455	-2.552	225, 201, 151
Kaempferol	$C_{15}H_{9}O_{6}$	7.80	11.5	285.0404	285.0399	-0.320	285, 257, 241, 229, 213, 185, 151, 107, 93
Isorhamnetin	$C_{16}H_{11}O_{7}$	7.98	11.5	315.0513	315.0499	0.775	300, 151
Kaempferol-3-*O-[6′′-O-*(trans-p-coumaroyl)-3''-*O*-acetyl*]-β-D-*glucopyranoside	$C_{32}H_{27}O_{14}$	8.10	19.5	635.1402	635.1401	-0.675	285
Trihydroxy octadecadienoic acid 1	$C_{18}H_{31}O_{5}$	8.11	3.5	327.2183	327.2177	1.903	n.a.
Pinellic acid	$C_{18}H_{33}O_{5}$	8.77	2.5	329.2329	329.2328	-1.237	229, 211, 171
3,5-Dihydroxy-hexadecanoic acid	$C_{16}H_{31}O_{4}$	9.08	1.5	287.2230	287.2217	0.722	269, 241
Trihydroxy octadecenoic acid	$C_{18}H_{33}O_{5}$	9.68	2.5	329.2335	329.2333	0.524	311, 293, 275
Araneophthalide	$C_{15}H_{17}O_{6}$	9.75	7.5	293.1035	293.1025	1.667	249 $[M-CO_2]^-$, 191 $[M-CO_2-C_3H_7O]^-$
5,3′,4′-trihydroxy-6,7,8-trimethoxyflavone	$C_{18}H_{15}O_{8}$	9.82	11.5	359.0774	359.0761	0.499	344, 331, 225, 210, 195
Unknown	$C_{17}H_{25}O_{4}$	10.76	5.5	293.1760	293.1747	0.673	n.a.
Phloroglucinol derivative analogue to 4-[3,5-dihydroxy-4-(2-methyl-1-oxopropyl)phenoxy]-2-methyl-2-butenoic acid methyl ester	$C_{16}H_{19}O_{6}$	11.07	7.5	307.1191	307.1176	1.200	263 $[M-H-CO_2]^-$, 194 $[M-H-C_6H_9O_2]^-$
Dihydroxy octadecadienoic acid	$C_{18}H_{31}O_{4}$	11.11	3.5	311.2227	311.2228	-0.105	293, 275
Unknown	$C_{20}H_{19}O_{6}$	11.29	11.5	355.1188	355.1176	0.249	n.a.
Acronyline	$C_{14}H_{17}O_{4}$	11.53	6.5	249.1134	249.1121	0.513	219 $[M-H-2CH_3]^-$, 180 [M-H-prenyl]$^-$
Octadecanedioic acid	$C_{18}H_{33}O_{4}$	11.68	2.5	313.2385	313.2373	0.087	295 $[M-H-H_2O]^-$
Prenyl phloroglucinol derivative	$C_{18}H_{17}O_{4}$	11.79	10.5	297.1131	297.1121	1.501	228 [M-H-prenyl]$^-$
Pyrone derivative	$C_{27}H_{27}O_{8}$	11.95	14.5	479.1716	479.1700	0.895	325, 313, 153 (pyrone fragment $C_8H_9O_3$)
Tetrahydrocurcumin	$C_{21}H_{25}O_{6}$	12.02	9.5	373.1660	373.1646	0.880	235 $[C_{13}H_{14}O_4]^-$, 193 $[C_{11}H_{13}O_3]^-$
Helenylonic acid (9-hydroxy-10E-octadecen-12-ynoic acid)	$C_{18}H_{29}O_{3}$	12.19	4.5	293.2125	293.2111	1.030	249, 197, 195, 185, 113
Unknown	$C_{25}H_{25}O_{6}$	12.25	13.5	421.1658	421.1646	0.399	n.a.
Phloroglucinol derivative equal to (E)-4-(3,5-dihydroxy-4-(2-methylbutanoyl)phenoxy)-2-methylbut-2-en-1-yl acetate	$C_{18}H_{23}O_{6}$	12.36	7.5	335.1502	335.1489	0.681	$[M-H-C_5H_9O]^-$: 251 ($C_{13}H_{15}O_5$) $[251-C_2OH_3]^-$: 209 ($C_{11}H_{13}O_4$)
Prenylated flavonoid	$C_{20}H_{17}O_{5}$	12.43	12.5	337.1082	337.1076	0.098	$[M-H-CO]^-$: 309, [M-H-prenyl]$^-$: 268
Hydroxy octadecadienoic acid	$C_{18}H_{31}O_{3}$	12.55	3.5	295.2281	295.2279	0.955	277
Prenylated polyphenol	$C_{26}H_{27}O_{6}$	12.57	13.5	435.1813	435.1808	0.065	$[M-H-C_5H_9]^-$: 366
Unknown	$C_{25}H_{23}O_{6}$	12.69	14.5	419.1505	419.1495	1.046	n.a.
Unknown	$C_{23}H_{29}O_{6}$	12.92	9.5	401.1974	401.1959	0.992	n.a.
Prenylated polyphenol	$C_{22}H_{23}O_{4}$	12.94	11.5	351.1605	351.1591	0.790	$[M-H-C_5H_9]^-$: 282 ($C_{17}H_{14}O_4$)
Pyrone derivative	$C_{25}H_{31}O_{8}$	12.98	10.5	459.2023	459.2013	-0.307	293, 153 (pyrone fragment $C_8H_9O_3$)
Unknown	$C_{22}H_{25}O_{6}$	13.04	10.5	385.1661	385.1646	1.164	n.a.

Unknown	$C_{26}H_{25}O_6$	13.08	14.5	433.1659	433.1646	0.596	n.a.
Prenylated phloroglucinol analogue to phenyl(2,4,6-trihydroxy-3,5-bis(3-methylbut-2-en-1-yl)phenyl)methanone	$C_{23}H_{25}O_4$	13.12	11.5	365.1760	365.1747	0.431	296 $[M\text{-}H\text{-}C_5H_9]^-$, 287 $[(M\text{-}2H\text{-}C_6H_5]^-$, 227 $[M\text{-}H\text{-}2C_5H_9]^-$
Pyrone derivative	$C_{21}H_{25}O_7$	13.16	9.5	389.1609	389.1595	0.729	223, 153 (pyrone fragment $C_8H_9O_3$)
Unknown	$C_{24}H_{31}O_6$	13.20	9.5	415.2129	415.2115	0.742	329, 303, 289, 277
Unknown	$C_{23}H_{27}O_6$	13.34	10.5	399.1816	399.1802	0.672	328, 301, 287, 273
Prenylated polyphenol with pyrone moiety	$C_{29}H_{41}O_5$	13.46	9.5	469.2960	469.2949	0.154	400 $[M\text{-}H\text{-}C_5H_9]^-$, 327, 301, 288, 273, 152 (pyrone fragment $C_7H_4O_4$)
Prenylated polyphenol with pyrone moiety	$C_{29}H_{41}O_6$	13.60	9.5	485.2913	485.2898	1.323	416 $[M\text{-}H\text{-}C_5H_9]^-$, 327, 301, 288, 273, 166 (pyrone fragment $C_8H_6O_4$)
Pyrone derivative	$C_{27}H_{25}O_7$	13.66	15.5	461.1608	461.1595	0.485	295, 153 (pyrone fragment $C_8H_9O_3$)
Prenylated polyphenol analogue to hyperbeanols and aristophenone A	$C_{33}H_{41}O_6$	13.70	13.5	533.2911	533.2898	0.408	515 $[M\text{-}H\text{-}H_2O]^-$, 501, 464 $[M\text{-}H\text{-}C_5H_9]^-$, 437, 419, 395 $[M\text{-}H\text{-}2C_5H_9]^-$, 387, 380, 368, 364, 321, 311, 296, 284, 275
Pyrone derivative	$C_{27}H_{27}O_7$	13.75	14.5	463.1762	463.1751	-0.035	297, 153 (pyrone fragment $C_8H_9O_3$)
Prenylated polyphenol with pyrone moiety	$C_{30}H_{43}O_5$	13.83	9.5	483.3116	483.3105	0.025	465, 439, 421, 414 $[M\text{-}H\text{-}C_5H_9]^-$, 397, 387, 371, 355, 345 $[M\text{-}H\text{-}2C_5H_9]^-$, 330, 315, 287, 275, 262, 219, 207, 194, 152 152 (pyrone fragment $C_7H_4O_4$)
Prenylated polyphenol	$C_{30}H_{43}O_6$	13.90	9.5	499.3065	499.3054	-0.045	481 $[M\text{-}H\text{-}H_2O]^-$, 467, 430 $[M\text{-}H\text{-}C_5H_9]^-$, 403, 385, 361, 346, 277, 249, 194
Cycloarzanol	$C_{24}H_{33}O_4$	14.01	8.5	385.2385	385.2373	0.071	341, 316, 301, 273, 248, 205, 180
Pyrone derivative	$C_{23}H_{25}O_7$	14.07	11.5	413.1610	413.1595	1.122	247, 153 (pyrone fragment $C_8H_9O_3$)
Italipyrone	$C_{22}H_{23}O_7$	14.25	11.5	399.1455	399.1444	1.387	399, 245, 233, 153 (pyrone fragment $C_8H_9O_3$), 109
Pyrone derivative	$C_{25}H_{35}O_4$	14.37	8.5	399.2542	399.2530	0.394	355, 330, 315, 287, 275, 262, 247, 219, 194, 152 (pyrone fragment $C_7H_4O_4$)
Pyrone derivative	$C_{23}H_{25}O_7$	14.67	11.5	413.1611	413.1595	1.267	369, 344, 329, 301, 276, 261, 247, 233, 153 (pyrone fragment $C_8H_9O_3$)
Pyrone derivative	$C_{28}H_{39}O_4$	14.73	9.5	439.2855	439.2843	0.175	395, 370, 327, 302, 301, 259, 234, 191, 166, 149, 138 (pyrone fragment $C_7H_6O_3$)
Pyrone derivative analogue to 22-methyl-22-ethyl-italipyron	$C_{24}H_{27}O_7$	15.04	11.5	427.1763	427.1751	0.102	383, 315, 301, 287, 275, 261, 247, 233, 221, 153 (pyrone fragment $C_8H_9O_3$)
Prenylated polyphenol with pyrone moiety	$C_{29}H_{41}O_4$	15.11	9.5	453.3008	453.2999	-0.426	409, 384 $[M\text{-}H\text{-}C_5H_9]^-$, 341, 316, 315, 273, 261, 248, 205, 193, 180, 152 (pyrone fragment $C_7H_4O_4$)
Prenylated polyphenol with pyrone moiety	$C_{30}H_{43}O_4$	15.45	9.5	467.3167	467.3156	-0.071	423, 398 $[M\text{-}H\text{-}C_5H_9]^-$, 355, 330, 329, 287, 275, 262, 219, 207, 194, 152 (pyrone fragment $C_7H_4O_4$)

Prenylated polyphenol	$C_{33}H_{41}O_4$	15.50	13.5	501.3006	501.2999	-0.864	457, 432 [M-H-C_5H_9]$^-$, 423, 389, 365, 363, 321, 309, 296, 295, 253, 241, 228
Oleylbitalin A	$C_{31}H_{45}O_4$	15.75	9.5	481.3321	481.3318	0.634	343 [M-H-$C_{10}H_{18}$]$^-$, 301 [M-H-$C_{13}H_{24}$]$^-$, 289 [M-H-$C_{14}H_{24}$]$^-$, 276 [M-H-$C_{15}H_{25}$]$^-$, 233 [M-H-$C_{18}H_{32}$]$^-$
Unknown	$C_{22}H_{25}O_8$	16.26		417.1551			277, 263, 251, 233, 221, 209, 193, 179, 167, 153 (pyrone fragment $C_8H_9O_3$)
Unknown	$C_{27}H_{27}O_8$	16.61	14.5	479.1711	479.1700	-0.065	
Arzanol analogue	$C_{23}H_{27}O_8$	16.68	10.5	431.1712	431.1700	0.206	
Pyrone derivative	$C_{27}H_{25}O_7$	16.72	15.5	461.1608	461.1600	1.674	417, 359, 315, 307, 295, 279, 223, 153 (pyrone fragment $C_8H_9O_3$)
Heliarzanol 2	$C_{24}H_{29}O_8$	16.79	10.5	445.1868	445.1862	0.020	445, 291, 279, 261
Arenol	$C_{21}H_{23}O_7$	16.86	10.5	387.1446	387.1444	0.625	261, 247, 235, 205, 166, 139 (pyrone fragment $C_7H_7O_3$)
Arzanol	$C_{22}H_{25}O_7$	17.05	10.5	401.1599	401.1600	1.661	247, 235, 205, 191, 166, 153 (pyrone fragment $C_8H_9O_3$)
Unknown	$C_{26}H_{25}O_7$	17.15	14.5	449.1607	449.1595	0.298	
3-Methylarzanol	$C_{23}H_{27}O_7$	17.22	10.5	415.1761	415.1757	0.329	415, 261, 249, 205, 193, 180, 153 (pyrone fragment $C_8H_9O_3$), 109
Unknown	$C_{27}H_{27}O_7$	17.34	14.5	463.1758	463.1757	0.964	309, 297, 227, 153 (pyrone fragment $C_8H_9O_3$)
6-*O*-desmethylauricepyron or arenol C analogue	$C_{24}H_{29}O_7$	17.47	10.5	429.1913	429.1913	0.112	153 (pyrone fragment $C_8H_9O_3$)
Auricepyron analogue	$C_{25}H_{31}O_7$	17.56	10.5	443.2072	443.2070	0.591	153 (pyrone fragment $C_8H_9O_3$)

Table S3. Annotated compounds in the methanol extract of *Helichrysum doerfleri* Rech. f. (HD) based on chromatographic and spectrometric data using UPLC-HRMS/MS in negative and positive ESI mode.

Compound	EC [M-H]$^-$ / [M+H]$^+$	IM	Rt (min)	RDBeq	Experimental *m/z*	Theoretical *m/z*	Δm (ppm)	MS/MS Fragments
Quinic acid	$C_7H_{11}O_6$	neg	0.86	2.5	191.0556	191.0561	-2.833	173, 171, 127
Malic acid	$C_4H_5O_5$	neg	1	2.5	133.0138	133.0142	-3.658	115
Disaccharide 1	$C_{12}H_{21}O_9$	neg	2.39	2.5	309.1181	309.1191	-3.253	263 [M-H-CH2O2]$^-$
Caffeoyl hexoside	$C_{15}H_{17}O_9$	neg	4	7.5	341.0866	341.0878	-3.592	179 [M-H-hexosyl]$^-$, 135 [M-H-hexosyl-CO_2]$^-$
Caffeoylquinic acid 3	$C_{16}H_{17}O_9$	neg	4.48	8.5	353.0863	353.0878	-4.348	191, 179, 173, 161, 135
Caffeic acid	$C_9H_7O_4$	neg	4.93	6.5	179.0344	179.035	-3.307	161, 135, 109
5-*O*-Feruloylquinic acid	$C_{17}H_{19}O_9$	neg	5.37	8.5	367.1021	367.1035	-3.665	191, 173, 134
unknown	$C_{10}H_{11}O_7$	neg	5.5	5.5	243.0502	243.051	-3.439	228 [M-H-CH_3]$^-$
Quercetin glycoside 2	$C_{21}H_{19}O_{12}$	neg	5.82	12.5	463.0865	463.0882	-3.756	301, 300, 273, 179, 151
Dicaffeoylquinic acid 2	$C_{25}H_{23}O_{12}$	neg	6.1	14.5	515.1178	515.1195	-3.299	353, 203, 191, 179, 173, 135
Kaempferol-3-*O*-glucoside	$C_{21}H_{19}O_{11}$	neg	6.19	12.5	447.0916	447.0933	-3.745	327, 285, 284, 255, 227, 211, 181, 175
Kaempferol-*O*-acetylhexoside	$C_{23}H_{21}O_{12}$	neg	6.45	13.5	489.1022	489.1038	-3.372	429, 327, 285, 284, 255, 213, 151
unknown	$C_9H_{15}O_4$	neg	6.68	2.5	187.0969	187.0976	-3.7	169 [M-H-H_2O]$^-$, 125 [M-H-H_2O-CO_2]$^-$
Kaempferol dihexoside 1	$C_{30}H_{25}O_{14}$	neg	6.84	18.5	609.1226	609.125	-3.872	447 [M-H-hexose]$^-$, 285 [M-H-2hexose]$^-$

Quercetin-7-*O*-(caffeoyl)-hexoside	$C_{30}H_{25}O_{15}$	neg	7.15	18.5	625.1174	625.1199	-3.94	463, 445, 323, 301, 273, 257, 179
Tiliroside	$C_{30}H_{25}O_{13}$	neg	7.25	18.5	593.1274	593.1301	-4.424	447, 307, 285
Quercetin	$C_{15}H_{9}O_{7}$	neg	7.52	11.5	301.0344	301.0354	-3.275	178, 151
Kaempferol-3-*O*-sophoroside-7-*O*-glucoside	$C_{32}H_{27}O_{15}$	neg	7.7	19.5	651.1334	651.1355	-3.353	609, 285
Kaempferol-3-*O*-[6''-*O*-(trans-p-coumaroyl)-3''-*O*-acetyl]-*β*-*D*-glucopyranoside	$C_{32}H_{27}O_{14}$	neg	8.19	19.5	635.138	635.1406	-4.139	285
C18 Trihydroxy fatty acid 2	$C_{18}H_{33}O_{5}$	neg	8.68	2.5	329.2321	329.2333	-3.819	229, 211, 171
3,5-Dihydroxy-hexadecanoic acid	$C_{16}H_{31}O_{4}$	neg	8.9	1.5	287.222	287.2228	-2.551	-
unknown	$C_{17}H_{15}O_{8}$	neg	9.48	10.5	347.0761	347.0772	-3.258	-
Prenylated polyphenol	$C_{22}H_{23}O_{4}$	neg	9.73	11.5	351.1589	351.1602	-3.538	282 $[M-H-prenyl]^-$
Methylated flavonoid 8	$C_{18}H_{17}O_{8}$	pos	10.26	10.5	361.0916	361.0918	-0.675	346 $[M+H-CH_3]^+$, 331 $[M+H-2CH_3]^+$
Terpene 1	$C_{17}H_{25}O_{4}$	neg	10.42	5.5	293.175	293.1758	-2.669	236, 221
Terpene 2	$C_{17}H_{25}O_{3}$	pos	10.85	5.5	277.1796	277.1798	-0.69	235, 221
Methylated flavonoid 17	$C_{18}H_{17}O_{7}$	pos	12.3	10.5	345.0963	345.0969	-1.563	330 $[M+H-CH_3]^+$, 315 $[M+H-2CH_3]^+$
C18 Hydroxy fatty acid	$C_{18}H_{31}O_{3}$	neg	12.84	3.5	295.2273	295.2279	-2.06	-
Linolenic acid	$C_{18}H_{31}O_{2}$	pos	13.24	3.5	279.2318	279.2319	-0.06	261, 223, 209
C17 Hydroxy fatty acid 2	$C_{17}H_{34}O_{5}$	pos	13.34	1	318.2401	318.2401	0.014	300 $[M+H-H_2O]^+$

Table S4. Annotated compounds in the methanol extract of *Helichrysum heldreichii* Boiss. (HH) based on chromatographic and spectrometric data using UPLC-HRMS/MS in negative and positive ESI mode.

Compound	EC $[M-H]^-$ / $[M+H]^+$	IM	Rt (min)	RDBeq	Experimental *m*/*z*	Theoretical *m*/*z*	Δm (ppm)	MS/MS Fragments
Glucoheptonic acid	$C_{7}H_{13}O_{8}$	neg	0.76	1.5	225.0608	225.0605	-3.691	179
Malic acid	$C_{4}H_{5}O_{5}$	neg	0.91	2.5	133.0138	133.0131	-3.508	115
Protocatechuic acid *O*-hexoside	$C_{13}H_{15}O_{9}$	neg	2.33	6.5	315.0711	315.0711	-3.318	153, 152, 109, 108
Disaccharide 1	$C_{12}H_{21}O_{9}$	neg	2.47	2.5	309.1183	309.118	-2.638	263 $[M-H-CH_2O_2]^-$
Caffeoylquinic acid 1	$C_{16}H_{17}O_{9}$	neg	2.7	8.5	353.0868	353.0867	-2.96	191, 179, 161, 135
Disaccharide 2	$C_{12}H_{21}O_{9}$	neg	3.46	2.5	309.1181	309.118	-3.35	263, 161
Caffeoylquinic acid 2	$C_{16}H_{17}O_{9}$	neg	3.65	8.5	353.0866	353.0867	-3.385	191, 179, 173, 161, 135
6-(*β*-*D*-glucopyranosyloxy)-7-hydroxy-2H-1-benzopyran-2-one (aesculin)	$C_{15}H_{15}O_{9}$	neg	4.12	8.5	339.0709	339.0711	-3.643	177
Quercetagetin-3,7-di-*O*-hexoside	$C_{27}H_{29}O_{18}$	neg	4.28	13.5	641.1334	641.1348	-4.02	479, 317
Caffeoylquinic acid 3	$C_{16}H_{17}O_{9}$	neg	4.47	8.5	353.0861	353.0867	-4.858	191, 179, 173, 161, 135
unknown	$C_{9}H_{13}O_{5}$	neg	4.8	3.5	201.0761	201.0757	-3.863	157, 139
Aesculetin	$C_{9}H_{5}O_{4}$	neg	4.86	7.5	177.0187	177.0182	-3.344	149, 133, 105, 89
Caffeic acid	$C_{9}H_{7}O_{4}$	neg	4.95	6.5	179.0344	179.0339	-3.474	135
unknown	$C_{18}H_{27}O_{9}$	neg	5.04	5.5	387.1648	387.165	-3.269	369 $[M-H-H_2O]^-$, 207, 163
Dicaffeoylquinic acid 1	$C_{25}H_{23}O_{12}$	neg	5.09	14.5	515.1179	515.1184	-3.182	353, 203, 191, 179, 173, 161
unknown	$C_{20}H_{31}O_{10}$	neg	5.16	5.5	431.1908	431.1912	-3.48	385, 223
5-*O*-Feruloylquinic acid	$C_{17}H_{19}O_{9}$	neg	5.31	8.5	367.1024	367.1024	-2.902	191, 173, 134
unknown	$C_{15}H_{21}O_{8}$	neg	5.71	5.5	329.1229	329.1231	-4.044	285 $[M-H-CO_2]^-$, 229, 179, 155

unknown	$C_{27}H_{35}O_{13}$	neg	5.75	10.5	567.2063	567.2072	-3.551	521, 359, 341, 329
Quercetin glycoside 2	$C_{21}H_{19}O_{12}$	neg	5.83	12.5	463.0864	463.0871	-3.82	301, 300, 179, 151
unknown	$C_{10}H_{11}O_5$	neg	5.9	5.5	211.0604	211.0601	-3.822	167 [M-H-CO_2]$^-$, 123 [167-CO_2]$^-$
Dicaffeoylquinic acid 3	$C_{25}H_{23}O_{12}$	neg	6.16	14.5	515.1171	515.1184	-4.6	353, 203, 191, 179, 173
unknown	$C_{25}H_{25}O_{11}$	neg	6.49	13.5	501.1385	501.1391	-3.541	339, 323, 177
Quercetin glycoside 3	$C_{21}H_{19}O_{12}$	neg	6.6	12.5	463.0866	463.0871	-3.82	301, 179, 151
unknown	$C_9H_{15}O_4$	neg	6.71	2.5	187.0969	187.0965	-3.86	169 [M-H-H_2O]$^-$, 125 [M-H-H_2O-CO_2]$^-$
Dicaffeoylquinic acid methyl ester	$C_{26}H_{25}O_{12}$	neg	6.8	14.5	529.1333	529.1341	-3.495	367, 353, 191, 179, 173
unknown	$C_{25}H_{23}O_{11}$	pos	6.83	14.5	499.1233	499.1235	-0.457	319, 163
Quercetin coumaroylglucoside	$C_{30}H_{25}O_{14}$	neg	6.9	18.5	609.1225	609.1239	-4.069	463, 301, 300, 255
unknown	$C_{15}H_{21}O_6$	pos	6.95	5.5	297.1331	297.1333	-0.487	279 [M+H-H_2O]$^+$, 223
unknown	$C_{22}H_{23}O_9$	neg	7.07	11.5	431.1333	431.1337	-3.306	387 [M-H-CO_2]$^-$, 179, 161
Quercetin-7-*O*-(caffeoyl)-hexoside	$C_{30}H_{25}O_{15}$	neg	7.09	18.5	625.1174	625.1188	-3.94	463, 445, 323, 301, 273, 257, 179
unknown	$C_{23}H_{25}O_{10}$	neg	7.19	18.5	461.1437	461.1442	-3.427	179, 161
Quercetin coumaroylglucoside analogue	$C_{30}H_{29}O_{15}$	neg	7.38	16.5	629.1483	629.1501	-4.678	463, 301
Kaempferol dihexoside 3	$C_{30}H_{25}O_{14}$	neg	7.45	18.5	609.1223	609.1239	-4.381	463, 447, 323, 285
Quercetin	$C_{15}H_9O_7$	neg	7.5	11.5	301.0342	301.0343	-3.973	283, 257, 179, 51, 107
unknown	$C_{23}H_{25}O_9$	neg	7.57	11.5	445.1487	445.1493	-3.944	401 [M-H-CO_2]$^-$, 179, 161
unknown	$C_{16}H_{19}O_6$	neg	7.62	7.5	307.1176	307.1176	-3.457	289 [M-H-H_2O]$^-$, 195, 167
unknown	$C_{26}H_{27}O_{12}$	neg	7.89	13.5	531.1489	531.1497	-3.52	487 [M-H-CO_2]$^-$, 341, 203
unknown	$C_{13}H_{15}O_3$	pos	7.94	6.5	219.1015	219.1016	-0.635	201 [M+H-H_2O]$^+$, 183, 159
unknown	$C_{16}H_{21}O_6$	pos	8.1	6.5	309.1329	309.1333	-1.083	291 [M+H-H_2O]$^+$
C18 Trihydroxy fatty acid 1	$C_{18}H_{31}O_5$	neg	8.26	3.5	327.2163	327.2166	-4.148	309, 291, 229, 211, 171
Kaempferol	$C_{15}H_9O_6$	neg	8.33	11.5	285.0394	285.0394	-3.723	285, 257, 241, 229, 213, 185, 151, 107, 93
unknown	$C_{15}H_{21}O_5$	pos	8.34	5.5	281.1381	281.1384	-0.926	263 [M+H-H_2O]$^+$, 235
unknown	$C_{19}H_{15}O_8$	neg	8.41	12.5	371.0757	371.0761	-4.206	353 [M-H-H_2O]$^-$, 327, 283
unknown	$C_{16}H_{23}O_6$	pos	8.46	5.5	311.1485	311.1489	-1.43	293 [M+H-H_2O]$^+$, 279, 265, 237, 155
Pinobanskin	$C_{15}H_{11}O_5$	neg	8.51	10.5	271.0604	271.0601	-2.976	253 [M-H-H_2O]$^-$, 225, 191, 151, 125
unknown	$C_{23}H_{21}O_8$	neg	8.55	13.5	425.1226	425.1231	-3.695	407 [M-H-H_2O]$^-$, 311
unknown	$C_{17}H_{23}O_7$	pos	8.56	6.5	339.1434	339.1438	-1.266	321 [M+H-H_2O]$^+$, 293
C18 Trihydroxy fatty acid 3	$C_{18}H_{33}O_5$	neg	8.59	2.5	329.2321	329.2323	-3.819	311 [M-H-H_2O]$^-$, 229, 211, 171
Dilignol analogue 1	$C_{20}H_{23}O_7$	neg	8.62	9.5	375.1437	375.1438	-3.162	357 [M-H-H_2O]$^-$, 331 [M-H-CO_2]$^-$
unknown	$C_{17}H_{13}O_6$	neg	8.8	11.5	313.0706	313.0707	-3.742	295 [M-H-H_2O]$^-$, 269
Dilignol analogue 2	$C_{20}H_{23}O_7$	neg	8.9	9.5	375.1433	375.1438	-4.388	357 [M-H-H_2O]$^-$, 331 [M-H-CO_2]$^-$
unknown	$C_{18}H_{13}O_8$	neg	9.02	12.5	357.0602	357.0605	-3.867	339 [M-H-H_2O]$^-$, 313, 298
unknown	$C_{20}H_{23}O_6$	pos	9.35	9.5	359.1487	359.1489	-0.654	341 [M+H-H_2O]$^+$, 313
unknown	$C_{21}H_{25}O_7$	neg	9.38	9.5	389.159	389.1595	-4.128	345 [M-H-CO_2]$^-$
unknown	$C_{19}H_{15}O_8$	neg	9.46	12.5	371.0757	371.0761	-4.125	353 [M-H-H_2O]$^-$, 327, 312
unknown	$C_{18}H_{15}O_8$	pos	9.54	11.5	359.0759	359.0761	-0.763	341 [M+H-H_2O]$^+$, 313
unknown	$C_{20}H_{21}O_7$	neg	9.56	10.5	373.1277	373.1282	-4.251	355 [M-H-H_2O]$^-$, 329
unknown	$C_{21}H_{25}O_7$	neg	9.64	9.5	389.159	389.1595	-4.128	345 [M-H-CO_2]$^-$
unknown	$C_{26}H_{31}O_8$	neg	9.72	11.5	471.2003	471.2013	-4.438	453 [M-H-H_2O]$^-$
unknown	$C_{24}H_{21}O_9$	neg	9.9	14.5	453.1172	453.118	-4.227	435 [M-H-H_2O]$^-$
unknown	$C_{18}H_{21}O_5$	neg	9.96	8.5	317.1382	317.1384	-4.09	273, 259, 167
unknown	$C_{19}H_{17}O_8$	pos	9.99	11.5	373.0915	373.0918	-0.815	355 [M+H-H_2O]$^+$, 327
unknown	$C_{26}H_{31}O_8$	neg	10.1	11.5	471.2005	471.2013	-4.12	453 [M-H-H_2O]$^-$
unknown	$C_{19}H_{25}O_8$	pos	10.14	7.5	381.1541	381.1544	-0.903	305, 293, 273

unknown	$C_{18}H_{21}O_5$	neg	10.22	8.5	317.138	317.1384	-4.563	273, 259, 167
unknown	$C_{26}H_{31}O_7$	pos	10.25	11.5	455.2064	455.2064	-0.153	437 $[M+H-H_2O]^+$, 335
unknown	$C_{25}H_{23}O_9$	neg	10.28	14.5	467.1333	467.1337	-3.116	449
unknown	$C_{24}H_{23}O_9$	pos	10.34	13.5	455.1333	455.1337	-0.854	437 $[M+H-H_2O]^+$, 313
Terpene 1	$C_{17}H_{25}O_4$	neg	10.5	5.5	293.1748	293.1747	-3.522	236, 221
unknown	$C_{25}H_{25}O_9$	pos	10.76	13.5	469.149	469.1493	-0.615	451 $[M+H-H_2O]^+$, 413, 327
Methylated flavonoid 11	$C_{16}H_{13}O_6$	pos	10.93	10.5	301.0705	301.0707	-0.547	286 $[M+H-CH_3]^+$
Methylated flavonoid 13	$C_{18}H_{17}O_7$	pos	11.17	10.5	345.0962	345.0969	-1.911	330 $[M+H-CH_3]^+$, 315 $[M+H-2CH_3]^+$
Methylated flavonoid 14	$C_{17}H_{15}O_6$	pos	11.31	10.5	315.0858	315.0863	-1.57	300 $[M+H-CH_3]^+$, 282 $[M+H-CH_3-H_2O]^+$
Methylated flavonoid 17	$C_{18}H_{17}O_7$	pos	12.37	10.5	345.0962	345.0969	-1.997	330 $[M+H-CH_3]^+$, 315 $[M+H-2CH_3]^+$, 297 $[M+H-2CH_3-H_2O]^+$
Methylated flavonoid 20	$C_{19}H_{19}O_7$	pos	12.88	10.5	359.1119	359.1125	-1.725	344 $[M+H-CH_3]^+$, 329 $[M+H-2CH_3]^+$, 311 $[M+H-2CH_3-H_2O]^+$
Triterpene 1	$C_{29}H_{40}O$	pos	15.6	5.5	413.3775	413.3778	-0.635	395 $[M+H-H_2O]^+$, 297, 255, 241
unknown	$C_{24}H_{35}O_3$	pos	16.26	7.5	371.258	371.2581	-0.273	353 $[M+H-H_2O]^+$, 167
Triterpene 2	$C_{29}H_{47}O$	pos	16.44	6.5	411.3618	411.3621	-1.266	393 $[M+H-H_2O]^+$, 327 $[M+H-C_6H_{12}]^+$, 309

Table S5. Annotated compounds in the methanol extract of *Helichrysum luteoalbum* (L.) Rchb. (HL) based on chromatographic and spectrometric data using UPLC-HRMS/MS in negative and positive ESI mode.

Compound	EC $[M-H]^-$ / $[M+H]^+$	IM	Rt (min)	RDBeq	Experimental *m/z*	Theoretical *m/z*	Δm (ppm)	MS/MS Fragments
Quinic acid	$C_7H_{11}O_6$	neg	0.86	2.5	191.0556	191.055	-2.519	173, 171, 127
Malic acid	$C_4H_5O_5$	neg	0.93	2.5	133.0138	133.0142	-3.056	115
Succinic acid	$C_4H_5O_4$	neg	1.36	2.5	117.0189	117.0193	-3.606	99, 73
Protocatechuic acid -*O*-hexoside	$C_{13}H_{15}O_9$	neg	2.27	6.5	315.0713	315.0722	-2.746	-
2,4-Dihydroxybenzoic acid	$C_7H_5O_4$	neg	3.07	5.5	153.0189	153.0193	-2.561	109
Caffeoylquinic acid 2	$C_{16}H_{17}O_9$	neg	3.56	8.5	353.0867	353.0867	-3.045	353, 203, 191, 179, 173, 161, 135
Caffeoyl hexoside	$C_{15}H_{17}O_9$	neg	4.21	7.5	341.0866	341.0867	-3.592	179, 135
Caffeoylquinic acid 3	$C_{16}H_{17}O_9$	neg	4.47	8.5	353.0865	353.0867	-3.81	353, 203, 191, 179, 173, 161, 135
Caffeic acid	$C_9H_7O_4$	neg	4.86	7.5	177.0188	177.0182	-2.836	161, 135
unknown	$C_{20}H_{31}O_{10}$	neg	5.13	5.5	431.191	431.1912	-2.992	385
5-*O*-Feruloylquinic acid	$C_{17}H_{19}O_9$	neg	5.34	8.5	367.1023	367.1024	-3.256	193, 191, 173, 134
unknown	$C_{20}H_{29}O_{12}$	neg	5.51	6.5	461.1649	461.1654	-3.447	415
Quercetin glycoside 2	$C_{21}H_{19}O_{12}$	neg	5.8	12.5	463.0866	463.0871	-3.367	301, 300, 179, 151
Kaempferol-3-*O*-glucoside	$C_{21}H_{19}O_{11}$	neg	5.84	12.5	447.0919	447.0922	-3.186	327, 285, 284, 283, 175
Dicaffeoylquinic acid 2	$C_{25}H_{23}O_{12}$	neg	6.01	14.5	515.1179	515.1184	-3.182	353, 203, 191, 179, 173, 161
Dicaffeoylquinic acid 3	$C_{25}H_{23}O_{12}$	neg	6.16	14.5	515.1174	515.1184	-4.134	353, 203, 191, 179, 173, 161
Kaempferol-7-*O*-hexoside	$C_{21}H_{19}O_{11}$	neg	6.25	12.5	447.0916	447.0922	-3.678	327, 285, 284
Apigenin-6-*C*-glucoside	$C_{21}H_{19}O_{10}$	neg	6.36	12.5	431.0969	431.0973	-3.433	431, 311, 269
unknown	$C_9H_{15}O_4$	neg	6.69	2.5	187.0969	187.0965	-3.593	169 $[M-H-H_2O]^-$, 125 $[M-H-H_2O-CO_2]^-$
Apigenin caffeoylhexoside 1	$C_{30}H_{25}O_{13}$	neg	7.05	18.5	593.1278	593.129	-3.8	323, 269
Kaempferol dihexoside 2	$C_{30}H_{25}O_{14}$	neg	7.19	18.5	609.1227	609.1239	-3.774	463, 447, 323, 285, 243, 241, 229
Apigenin caffeoylhexoside 2	$C_{30}H_{25}O_{13}$	neg	7.3	18.5	593.1279	593.129	-3.699	323, 269

Luteolin	$C_{15}H_9O_6$	neg	7.45	11.5	285.0392	285.0394	-4.6	175, 151, 133
Apigenin	$C_{15}H_9O_5$	neg	8.18	11.5	269.0443	269.0444	-4.82	225, 201, 151
C18 Trihydroxy fatty acid 2	$C_{18}H_{33}O_5$	neg	8.65	2.5	329.2321	329.2323	-3.91	314, 311, 309 $[M-H_2O]^-$, 293, 291 $[M-2H_2O]^-$, 229, 211, 183, 171
Methylated flavonoid 3	$C_{19}H_{17}O_9$	neg	9.3	11.5	389.0864	389.0867	-3.535	374 $[M-H-CH_3]^-$
Methylated flavonoid 4	$C_{19}H_{19}O_9$	pos	9.75	10.5	391.1024	391.1024	0.004	376 $[M+H-CH_3]^+$, 361 $[M+H-2CH_3]^+$, 343 $[M+H-2CH_3-H_2O]^+$
Methylated flavonoid 7	$C_{19}H_{19}O_8$	pos	10.25	10.5	375.1074	375.1074	-0.224	360 $[M+H-CH_3]^+$, 345 $[M+H-2CH_3]^+$
Methylated flavonoid 9	$C_{19}H_{19}O_8$	pos	10.73	10.5	375.1068	375.1074	-1.77	360 $[M+H-CH_3]^+$, 345 $[M+H-2CH_3]^+$, 327 $[M+H-2CH_3-H_2O]^+$
Methylated flavonoid 10	$C_{20}H_{21}O_9$	pos	10.9	10.5	405.1174	405.118	-1.502	390 $[M+H-CH_3]^+$, 375 $[M+H-2CH_3]^+$, 372, 357 $[M+H-2CH_3-H_2O]^+$
Methylated flavonoid 12	$C_{17}H_{15}O_6$	pos	10.96	10.5	315.0861	315.0863	-0.618	300 $[M+H-CH_3]^+$, 285 $[M+H-2CH_3]^+$
Methylated flavonoid 13	$C_{18}H_{17}O_7$	pos	11	10.5	345.0965	345.0969	-1.099	330 $[M+H-CH_3]^+$, 315 $[M+H-2CH_3]^+$
Methylated flavonoid 16	$C_{21}H_{23}O_9$	pos	12.03	10.5	419.1332	419.1337	-1.142	404 $[M+H-CH_3]^+$, 389 $[M+H-2CH_3]^+$, 371 $[M+H-2CH_3-H_2O]^+$
Methylated flavonoid 17	$C_{18}H_{17}O_7$	pos	12.29	10.5	345.0964	345.0969	-1.36	330 $[M+H-CH_3]^+$, 315 $[M+H-2CH_3]^+$
Diterpene	$C_{18}H_{29}O_2$	pos	12.46	4.5	277.2161	277.2162	-0.529	259 $[M+H-H_2O]^+$, 241, 195, 163, 149, 135, 121, 107, 93, 81
Methylated flavonoid 18	$C_{18}H_{17}O_6$	pos	12.56	10.5	329.1016	329.102	-1.047	314 $[M+H-CH_3]^+$, 299 $[M+H-2CH_3]^+$
Methylated flavonoid 19	$C_{20}H_{21}O_8$	pos	12.8	10.5	389.1229	389.1231	-0.524	374 $[M+H-CH_3]^+$, 359 $[M+H-2CH_3]^+$, 341 $[M+H-2CH_3-H_2O]^+$
Oxygenated aliphatic hydrocarbon	$C_{17}H_{34}O_5$	pos	13.28	1	318.2399	318.2401	-0.552	300
Auricepyron analogue	$C_{25}H_{33}O_7$	pos	13.7	9.5	445.2217	445.2221	-0.763	389, 361, 291, 279, 223
Oxygenated aliphatic hydrocarbon	$C_{17}H_{36}O_5$	pos	14.15	0	320.2556	320.2557	-0.549	194 $[M+H-C_9H_{18}]^+$
unknown	$C_{26}H_{39}O_8$	pos	14.9	7.5	479.2618	479.2639	-4.454	367, 318
Lupulone	$C_{26}H_{37}O_4$	neg	16.4	8.5	413.2686	413.2697	-2.766	369 $[M-H-CO_2]^-$, 344 $[M-H-prenyl]^-$, 301 $[M-H-prenyl-C_3H_7]^-$, 289, 276, 233, 208, 152
unknown	$C_{26}H_{37}O_5$	neg	16.65	8.5	429.2631	429.2646	-3.698	411, 333, 263
unknown	$C_{25}H_{31}O_7$	neg	16.84	10.5	443.206	443.2075	-3.467	289, 277

Table S6. Annotated compounds in the methanol extract of *Helichrysum orientale* (L.) Gaertn. (HO) based on chromatographic and spectrometric data using UPLC-HRMS/MS in negative and positive ESI mode.

Compound	EC $[M-H]^-$ / $[M+H]^+$	IM	Rt (min)	RDBeq	Experimental *m/z*	Theoretical *m/z*	Δm (ppm)	MS/MS Fragments
Hexose	$C_6H_{11}O_6$	neg	0.76	1.5	179.0555	179.0561	-3.247	161, 141, 135, 117, 99, 87
Malic acid	$C_4H_5O_5$	neg	0.94	2.5	133.0137	133.0142	-3.883	-
Syringic acid 4-*O*-*β*-*D*-glucopyranoside	$C_{15}H_{19}O_{10}$	neg	3.14	6.5	359.0971	359.0984	-3.425	-
Caffeoyl hexoside	$C_{15}H_{17}O_9$	neg	3.94	7.5	341.0865	341.0878	-3.768	179, 135
Caffeoylquinic acid 3	$C_{16}H_{17}O_9$	neg	4.46	8.5	353.0863	353.0878	-4.263	191, 179, 173, 161, 135, 127
Caffeic acid	$C_9H_7O_4$	neg	4.88	6.5	179.0343	179.035	-3.642	161, 135
Dicaffeoylquinic acid 1	$C_{25}H_{23}O_{12}$	neg	5.08	14.5	515.1176	515.1195	-3.784	353, 203, 191, 179, 173, 161
unknown	$C_{20}H_{31}O_{10}$	neg	5.13	5.5	431.1909	431.1923	-3.271	-
5-*O*-Feruloylquinic acid	$C_{17}H_{19}O_9$	neg	5.34	8.5	367.1023	367.1035	-3.256	193, 191, 173, 134
Naringenin-*O*-hexoside 1	$C_{21}H_{21}O_{10}$	neg	5.62	11.5	433.1122	433.114	-4.248	313, 271, 151
Naringenin-*O*-hexoside 2	$C_{21}H_{21}O_{10}$	neg	5.72	11.5	433.1119	433.114	-4.803	313, 271, 151
Dicaffeoylquinic acid 2	$C_{25}H_{23}O_{12}$	neg	6.07	14.5	515.1175	515.1195	-3.901	353, 203, 191, 179, 173, 161

Kaempferol-3-*O*-glucoside	$C_{21}H_{19}O_{11}$	neg	6.18	12.5	447.0911	447.0933	-4.819	327, 285, 284, 283, 255, 227, 211, 181, 175
unknown	$C_9H_{15}O_4$	neg	6.66	2.5	187.0969	187.0976	-3.86	169 $[M-H-H_2O]^-$, 125 $[M-H-H_2O-CO_2]^-$
Naringenin-*O*-hexoside 3	$C_{21}H_{21}O_{10}$	neg	6.76	11.5	433.112	433.114	-4.664	313, 271, 151
Tiliroside	$C_{30}H_{25}O_{13}$	neg	7.24	18.5	593.1276	593.1301	-4.12	447, 307, 285, 257, 241, 229, 213
Naringenin	$C_{15}H_{11}O_5$	neg	8.18	10.5	271.0602	271.0612	-3.751	177, 151, 119, 107, 93, 83
Kaempferol	$C_{15}H_9O_6$	neg	8.3	11.5	285.0393	285.0405	-3.934	285, 257, 241, 229, 213, 185, 151, 107
C18 Trihydroxy fatty acid 2	$C_{18}H_{33}O_5$	neg	8.64	2.5	329.2321	329.2333	-3.728	311 $[M-H_2O]^-$, 293 $[M-2H_2O]^-$, 275 $[M-3H_2O]^-$, 229, 211, 183, 171
Terpene 1	$C_{17}H_{25}O_4$	neg	10.4	5.5	293.1748	293.1758	-3.624	236, 221
Terpene 2	$C_{17}H_{25}O_3$	pos	10.55	5.5	277.1798	277.1798	-0.148	235, 221
Methylated flavonoid 15	$C_{18}H_{17}O_7$	pos	12	10.5	345.0966	345.0969	-0.838	330 $[M+H-CH_3]^+$, 315 $[M+H-2CH_3]^+$
C17 Hydroxy fatty acid 2	$C_{17}H_{34}O_5$	pos	13	1	318.2404	318.2401	1.082	300 $[M+H-H_2O]^+$
unknown prenylated	$C_{26}H_{37}O_5$	neg	16.21	8.5	429.263	429.2646	-3.838	-

Table S7. Annotated compounds in the methanol extract of *Helichrysum plicatum* DC. (HP) based on chromatographic and spectrometric data using UPLC-HRMS/MS in negative ESI mode.

Compound	EC $[M-H]^-$ / $[M+H]^+$	IM	Rt (min)	RDBeq	Experimental *m/z*	Theoretical *m/z*	Δm (ppm)	MS/MS Fragments
Glucoheptonic acid	$C_7H_{13}O_8$	neg	0.76	1.5	225.0607	225.0616	-3.735	179
Quinic acid	$C_7H_{11}O_6$	neg	0.9	2.5	191.0554	191.0561	-3.723	173, 171, 155, 127, 111, 109
Malic acid	$C_4H_5O_5$	neg	0.99	2.5	133.0137	133.0142	-3.733	115
Caffeoyl glucose	$C_{15}H_{17}O_9$	neg	1.62	7.5	341.0868	341.0878	-2.889	179, 135
Protocatechuic acid-*O*-hexoside	$C_{13}H_{15}O_9$	neg	2.24	6.5	315.0713	315.0722	-2.841	153, 152, 109, 108
Caffeoylquinic acid 1	$C_{16}H_{17}O_9$	neg	2.64	8.5	353.0865	353.0878	-3.555	191, 179, 173, 161, 135
Caffeoylquinic acid 2	$C_{16}H_{17}O_9$	neg	3.58	8.5	353.0867	353.0878	-3.13	191, 179, 173, 161, 135
Caffeoyl hexoside	$C_{15}H_{17}O_9$	neg	3.84	7.5	341.0866	341.0878	-3.416	179, 135
Caffeoylquinic acid 3	$C_{16}H_{17}O_9$	neg	4.47	8.5	353.0862	353.0878	-4.518	191, 179, 173, 161, 135
Caffeic acid	$C_9H_7O_4$	neg	4.91	6.5	179.0343	179.035	-3.698	161, 135
Dicaffeoylquinic acid 1	$C_{25}H_{23}O_{12}$	neg	5.09	14.5	515.1178	515.1195	-3.299	353, 335, 203, 191, 179, 173, 161, 135
Isovitexin 2''-*O*-*β*-*D*-glucoside HCOOH adduct	$C_{28}H_{31}O_{17}$	neg	5.14	13.5	639.1541	639.1567	-4.009	593, 431, 477, 311
5-*O*-Feruloylquinic acid	$C_{17}H_{19}O_9$	neg	5.38	8.5	367.1024	367.1035	-2.902	193, 191, 173, 134
Naringenin-*O*-hexoside 1	$C_{21}H_{21}O_{10}$	neg	5.62	11.5	433.112	433.114	-4.595	313, 271, 151
Naringenin-*O*-hexoside 2	$C_{21}H_{21}O_{10}$	neg	5.77	11.5	433.1119	433.114	-4.803	313, 271, 151
5,6,4'-Trihydroxy-3'-methoxyflavone-7-*O*-*β*-glucoside	$C_{22}H_{21}O_{12}$	neg	6	12.5	477.102	477.1038	-3.834	433, 315
Dicaffeoylquinic acid 2	$C_{25}H_{23}O_{12}$	neg	6.13	14.5	515.117	515.1195	-4.852	353, 335, 203, 191, 179, 173, 161
Apigenin-6-*C*-glucoside	$C_{21}H_{19}O_{10}$	neg	6.35	12.5	431.0966	431.0984	-4.199	431, 311, 269
Naringenin-*O*-hexoside 3	$C_{21}H_{21}O_{10}$	neg	6.78	11.5	433.1122	433.114	-4.179	313, 271, 151
Luteolin	$C_{15}H_9O_6$	neg	7.42	11.5	285.0394	285.0405	-3.618	175, 151, 133
unknown	$C_{25}H_{23}O_{13}$	neg	7.58	14.5	531.1125	531.1144	-3.509	471, 327, 285, 284, 255
Dihydrokaempferol	$C_{15}H_{11}O_6$	neg	7.65	10.5	287.0552	287.0561	-3.035	269, 243
Methylated flavonoid 1	$C_{18}H_{15}O_9$	neg	7.85	11.5	375.0704	375.0722	-4.68	360 $[M-H-CH_3]^-$
Apigenin	$C_{15}H_9O_5$	neg	8.14	11.5	269.0443	269.0455	-4.708	225, 201, 151
Methylated flavonoid 2	$C_{18}H_{15}O_8$	neg	8.68	11.5	359.0757	359.0772	-4.179	344 $[M-H-CH_3]^-$
unknown	$C_{21}H_{25}O_5$	neg	8.91	9.5	357.1696	357.1707	-3.324	313 $[M-H-CO_2]^-$, 225, 145, 119
unknown	$C_{23}H_{25}O_6$	neg	9.09	11.5	397.1641	397.1657	-3.957	379 $[M-H-H_2O]^-$, 255, 241
unknown	$C_{14}H_{11}O_4$	neg	9.42	9.5	243.0652	243.0663	-4.452	228 $[M-H-CH_3]^-$, 227 $[M-2H-CH_3]^-$, 165, 145

unknown	$C_{23}H_{27}O_6$	neg	9.64	10.5	399.1796	399.1813	-4.363	381 $[M-H-H_2O]^-$, 355, 321, 253, 228, 185, 143
unknown	$C_{23}H_{25}O_6$	neg	9.85	11.5	397.1643	397.1657	-3.404	379 $[M-H-H_2O]^-$, 241, 161
unknown	$C_{21}H_{23}O_4$	neg	9.99	10.5	339.159	339.1602	-3.487	295, 277, 237, 200, 157, 119
unknown	$C_{23}H_{25}O_6$	neg	10.13	11.5	397.1643	397.1657	-3.479	379 $[M-H-H_2O]^-$, 339, 309, 251, 241, 145
unknown	$C_{21}H_{23}O_4$	neg	10.4	10.5	339.1589	339.1602	-3.87	321, 295, 225, 157, 145, 119
unknown	$C_{23}H_{25}O_5$	neg	10.91	11.5	381.1693	381.1707	-3.77	363 $[M-H-H_2O]^-$, 337 $[M-H-CO_2]^-$, 282, 253, 241, 229, 199, 145, 119
unknown	$C_{23}H_{25}O_5$	neg	11.1	11.5	381.1692	381.1707	-4.006	337 $[M-H-CO_2]^-$, 303, 267, 235, 199, 145, 119
unknown	$C_{23}H_{25}O_6$	neg	11.29	11.5	397.1645	397.1657	-2.875	379 $[M-H-H_2O]^-$, 244
unknown	$C_{23}H_{25}O_5$	neg	11.88	11.5	381.1696	381.1707	-2.878	363 $[M-H-H_2O]^-$, 255, 241, 229, 197, 163
unknown	$C_{21}H_{23}O_3$	neg	12.8	10.5	323.1643	323.1653	-3.057	279 $[M-H-CO_2]^-$
unknown	$C_{23}H_{25}O_4$	neg	13.77	11.5	365.1742	365.1758	-4.58	321, 287, 241, 228, 201, 145
Helicerastripyrone	$C_{27}H_{33}O_7$	neg	16.17	11.5	469.2215	469.2232	-3.637	315, 303, 153
unknown	$C_{31}H_{33}O_7$	neg	16.7	15.5	517.2213	517.2232	-3.609	377, 365
3-[[3-acetyl-5-(3,7-dimethyl-2,6-octadienyl)-2,4,6-trihydroxyphenyl]methyl]-4-hydroxy-5-methyl-6-propyl-2H-pyran-2-one **or** 3-[[3-(3,7-dimethyl-2,6-octadien-1-yl)-2,4,6-trihydroxy-5-(2-methyl-1-oxopropyl)phenyl]methyl]-4-hydroxy-5,6-dimethyl-2H-pyran-2-one	$C_{28}H_{35}O_7$	neg	16.75	11.5	483.237	483.2388	-3.842	329, 317
unknown	$C_{32}H_{35}O_7$	neg	16.97	15.5	531.2364	531.2388	-4.587	487 $[M-H-CO_2]^-$, 377, 365
3-[[3-(3,7-dimethyl-2,6-octadien-1-yl)-2,4,6-trihydroxy-5-(2-methyl-1-oxobutyl)phenyl]methyl]-4-hydroxy-5,6-dimethyl-2H-pyran-2-one	$C_{29}H_{37}O_7$	neg	17.43	11.5	497.2525	497.2545	-3.915	343, 331

Table S8. Annotated compounds in the methanol extract of *Helichrysum sibthorpii* Rouy (HS) based on chromatographic and spectrometric data using UPLC-HRMS/MS in negative ESI mode.

Compound	EC $[M-H]^-$ / $[M+H]^+$	IM	Rt (min)	RDBeq	Experimental *m/z*	Theoretical *m/z*	Δm (ppm)	MS/MS Fragments
Quinic acid	$C_7H_{11}O_6$	neg	0.85	2.5	191.0555	191.0561	-3.304	-
Malic acid	$C_4H_5O_5$	neg	0.97	2.5	133.0138	133.0142	-3.432	115
unknown	$C_6H_{11}O_4$	neg	1.46	1.5	147.0659	147.0663	-2.394	129 $[M-H-H_2O]^-$
Protocatechuic acid-*O*-hexoside	$C_{13}H_{15}O_9$	neg	2.3	6.5	315.0713	315.0722	-2.841	153, 152, 109, 108
Caffeoylquinic acid 1	$C_{16}H_{17}O_9$	neg	2.65	8.5	353.0869	353.0878	-2.536	191, 179, 173, 161, 135
Caffeoylquinic acid 2	$C_{16}H_{17}O_9$	neg	3.6	8.5	353.087	353.0878	-2.337	191, 179, 173, 161, 135
Caffeoyl hexoside	$C_{15}H_{17}O_9$	neg	3.9	7.5	341.0867	341.0878	-3.241	179, 135
1-*O*-Protocatechuyl-*β*-*D*-xylopyranose (uralenneoside)	$C_{12}H_{13}O_8$	neg	4	6.5	285.0609	285.0616	-2.598	153, 109
Caffeoylquinic acid 3	$C_{16}H_{17}O_9$	neg	4.48	8.5	353.0863	353.0878	-4.263	191, 179, 173, 161, 135
Caffeic acid	$C_9H_7O_4$	neg	4.9	6.5	179.0345	179.035	-2.804	161, 135
Dicaffeoylquinic acid 1	$C_{25}H_{23}O_{12}$	neg	5.1	14.5	515.1182	515.1195	-2.464	353, 203, 191, 179, 173, 161
5-*O*-Feruloylquinic acid	$C_{17}H_{19}O_9$	neg	5.34	8.5	367.1023	367.1035	-3.175	191, 173, 134

unknown	$C_{10}H_{11}O_7$	neg	5.48	5.5	243.0502	243.051	-3.563	228 [M-H-CH_3]$^-$
unknown	$C_{20}H_{29}O_{12}$	neg	5.57	6.5	461.1652	461.1664	-2.731	415
Quercetin glycoside 2	$C_{21}H_{19}O_{12}$	neg	5.79	12.5	463.0867	463.0882	-3.302	301
unknown	$C_{25}H_{39}O_{13}$	neg	6.04	6.5	547.238	547.2396	-2.877	501, 353, 311, 293, 221
Dicaffeoylquinic acid 2	$C_{25}H_{23}O_{12}$	neg	6.13	14.5	515.1171	515.1195	-4.6	353, 203, 191, 179, 173, 161
unknown	$C_9H_{15}O_4$	neg	6.67	2.5	187.097	187.0976	-3.219	169 [M-H-H_2O]$^-$, 125 [M-H-H_2O-CO_2]$^-$
Oxododecanedioic acid	$C_{12}H_{19}O_5$	neg	6.78	3.5	243.123	243.1238	-3.278	225, 181
unknown	$C_{14}H_{17}O_5$	neg	6.94	6.5	265.1074	265.1081	-2.742	247 [M-H-H_2O]$^-$, 207, 193
Coumarin analogue 1	$C_{15}H_{17}O_7$	neg	7.04	7.5	309.0968	309.098	-3.805	291 [M-H-H_2O]$^-$, 265, 247
Tiliroside	$C_{30}H_{25}O_{13}$	neg	7.26	18.5	593.1282	593.1301	-3.193	447, 307, 285
unknown	$C_{11}H_{13}O_5$	neg	7.59	5.5	225.0761	225.0768	-3.407	207 [M-H-H_2O]$^-$, 180
unknown	$C_{16}H_{21}O_7$	neg	7.79	6.5	325.1283	325.1293	-3.003	293, 281, 249, 237, 221, 205, 171, 139
unknown	$C_{16}H_{19}O_7$	neg	7.86	7.5	323.1127	323.1136	-2.774	279 [M-H-CO_2]$^-$
unknown	$C_{14}H_{19}O_5$	neg	7.87	5.5	251.1281	251.1289	-3.195	207 [M-H-CO_2]$^-$, 179, 167
unknown	$C_{12}H_{13}O_6$	neg	8	6.5	253.0709	253.0718	-3.325	235 [M-H-H_2O]$^-$, 209 [M-H-CO_2]$^-$
unknown prenylated	$C_{20}H_{27}O_5$	neg	8.09	7.5	347.1852	347.1864	-3.333	278 [M-H-prenyl]$^-$
C18 Trihydroxy fatty acid 1	$C_{18}H_{31}O_5$	neg	8.26	3.5	327.2164	327.2177	-3.873	309 [M-H_2O]$^-$, 291 [M-2H_2O]$^-$, 273 [M-3H_2O]$^-$, 239, 229, 211, 183, 171
Coumarin analogue 2	$C_{15}H_{17}O_7$	neg	8.36	7.5	309.0971	309.098	-2.899	291 [M-H-H_2O]$^-$, 265, 247, 237, 181, 139
unknown phloroglucinol	$C_{15}H_{21}O_5$	neg	8.48	5.5	281.1384	281.1394	-3.724	263 [M-H-H_2O]$^-$, 249, 237, 179, 171, 127, 109
unknown	$C_{17}H_{21}O_7$	neg	8.5	7.5	337.128	337.1293	-3.904	319 [M-H-H_2O]$^-$, 293 [M-H-CO_2]$^-$, 235, 195, 129
Pyrone derivative 1	$C_{17}H_{23}O_7$	neg	8.51	6.5	339.1434	339.1449	-4.412	307, 295 [M-H-CO_2]$^-$, 279, 263, 237, 235, 205, 153 [$C_8H_9O_3$]$^-$
C18 Trihydroxy fatty acid 2	$C_{18}H_{33}O_5$	neg	8.7	2.5	329.2322	329.2333	-3.546	311 [M-H_2O]$^-$, 293 [M-2H_2O]$^-$, 275 [M-3H_2O]$^-$, 229, 211, 183, 171
unknown	$C_{16}H_{21}O_5$	neg	8.75	6.5	293.1387	293.1394	-2.548	275 [M-H-H_2O]$^-$, 235, 221, 191, 165, 141
unknown	$C_{13}H_{15}O_6$	neg	8.98	6.5	267.0864	267.0874	-3.937	249 [M-H-H_2O]$^-$, 223 [M-H-CO_2]$^-$, 193, 179
unknown	$C_{15}H_{19}O_5$	neg	9.02	6.5	279.1228	279.1238	-3.464	235 [M-H-CO_2]$^-$, 191
unknown	$C_{18}H_{23}O_7$	neg	9.13	7.5	351.1438	351.1449	-3.122	307 [M-H-CO_2]$^-$, 289 [307-H_2O]$^-$
Araneophthalide	$C_{15}H_{17}O_6$	neg	9.41	7.5	293.1023	293.1031	-2.496	249 [M-H-CO_2]$^-$
unknown	$C_{13}H_{17}O_5$	neg	9.6	5.5	253.1073	253.1081	-3.149	235 [M-H-H_2O]$^-$, 208, 183, 165, 141, 109
unknown	$C_{19}H_{23}O_8$	neg	9.78	8.5	379.1385	379.1398	-3.484	361 [M-H-H_2O]$^-$, 335 [M-H-CO_2]$^-$, 307 [335-CO]$^-$, 267, 209, 165
Methylated flavonoid 5	$C_{18}H_{15}O_8$	neg	9.82	11.5	359.0762	359.0772	-2.815	344 [M-H-CH_3]$^-$, 331, 225, 210, 194
unknown	$C_{23}H_{29}O_8$	neg	9.9	9.5	433.1854	433.1868	-3.165	415 [M-H-H_2O]$^-$, 405 [M-H-CO]$^-$, 377, 347, 319, 279, 221, 177, 135
Pyrone derivative 2	$C_{14}H_{17}O_5$	neg	10.02	6.5	265.1074	265.1081	-2.968	247 [M-H-H_2O]$^-$, 233, 195, 180, 153 [$C_8H_9O_3$]$^-$
unknown	$C_{17}H_{21}O_7$	neg	10.3	7.5	337.1293	337.1293	-2.985	319 [M-H-H_2O]$^-$, 293 [M-H-CO_2]$^-$, 275 [293-H_2O]$^-$, 221, 181, 141
unknown	$C_{19}H_{27}O_6$	neg	10.39	6.5	351.1804	351.1813	-2.539	333 [M-H-H_2O]$^-$, 279
unknown	$C_{24}H_{31}O_8$	neg	10.44	9.5	447.2015	447.2024	-2.149	429 [M-H-H_2O]$^-$, 419 [M-H-CO_2]$^-$, 333, 293
unknown	$C_{19}H_{25}O_4$	neg	10.54	7.5	317.175	317.1758	-2.656	289 [M-H-H_2O]$^-$, 248, 220, 180, 152
unknown	$C_{18}H_{23}O_7$	neg	10.92	7.5	351.144	351.1449	-2.695	307 [M-H-CO_2]$^-$, 289 [307-H_2O]$^-$, 235, 181, 125
unknown	$C_{17}H_{21}O_6$	neg	11	7.5	321.1336	321.1344	-2.434	303 [M-H-H_2O]$^-$, 277 [M-H-CO_2]$^-$, 259 [277-H_2O]$^-$, 233, 207, 165, 111
unknown	$C_{20}H_{29}O_6$	neg	11.12	6.5	365.1961	365.197	-2.333	347 [M-H-H_2O]$^-$, 293
unknown prenylated	$C_{20}H_{27}O_4$	neg	11.5	7.5	331.1905	331.1915	-2.907	303 [M-H-H_2O]$^-$, 262 [M-H-prenyl]$^-$, 234 [262-CO]$^-$, 194, 166

unknown	$C_{14}H_{17}O_4$	neg	11.63	6.5	249.1123	249.1132	-3.582	234 $[M-H-CH_3]^-$, 207, 191, 179, 166, 151, 125
unknown	$C_{15}H_{19}O_4$	neg	12.56	6.5	263.1281	263.1289	-2.821	248 $[M-H-CH_3]^-$, 207, 205, 193, 180, 151
Arzanol	$C_{22}H_{25}O_7$	neg	12.85	10.5	401.1595	401.1606	-2.558	247, 235, 205, 191, 166, 153 $[C_8H_9O_3]^-$
unknown	$C_{16}H_{21}O_4$	neg	13.16	6.5	277.1436	277.1445	-3.401	262 $[M-H-CH_3]^-$, 259 $[M-H-H_2O]^-$, 233 $[M-H-CO_2]^-$, 219, 207, 194, 179
Plicatipyrone analogue 2	$C_{22}H_{25}O_8$	neg	13.33	10.5	417.1545	417.1555	-2.28	263, 251, 153 $[C_8H_9O_3]^-$
Pyrone derivative 3	$C_{25}H_{31}O_8$	neg	13.42	10.5	459.2009	459.2024	-3.421	293, 153 $[C_8H_9O_3]^-$
unknown	$C_{18}H_{21}O_5$	neg	13.58	8.5	317.1385	317.1394	-3.112	299 $[M-H-H_2O]^-$, 289 $[M-H-CO]^-$, 248
3-Methylarzanol	$C_{23}H_{27}O_7$	neg	13.66	10.5	415.1752	415.1762	-2.544	261, 249, 247, 235, 205, 193, 180, 153
unknown	$C_{17}H_{23}O_4$	neg	13.74	6.5	291.1594	291.1602	-2.619	279 $[M-H-CH_3]^-$, 273 $[M-H-H_2O]^-$, 233, 221, 208, 207, 179
3-Methylarzanol isomer	$C_{23}H_{27}O_7$	neg	14	10.5	415.175	415.1762	-2.906	261, 249, 205, 193, 180, 153
unknown	$C_{24}H_{31}O_6$	neg	14.08	9.5	415.2115	415.2126	-2.702	397 $[M-H-H_2O]^-$, 346, 329, 303, 289, 277, 261, 249
unknown	$C_{23}H_{27}O_6$	neg	14.21	10.5	399.1804	399.1813	-2.309	355 $[M-H-CO_2]^-$, 287
6-*O*-desmethylauricepyron or arenol C analogue	$C_{24}H_{29}O_7$	neg	14.5	10.5	429.1906	429.1919	-3.091	275, 263, 247, 153
Auricepyron analogue 1	$C_{25}H_{31}O_7$	neg	15.2	10.5	443.2057	443.2075	-4.144	289, 277, 261, 193, 153 $[C_8H_9O_3]^-$
Pyrone derivative 4	$C_{25}H_{31}O_8$	neg	15.65	10.5	459.2004	459.2024	-4.423	305, 293, 153 $[C_8H_9O_3]^-$
unknown prenylated	$C_{25}H_{35}O_4$	neg	15.94	8.5	399.2527	399.2541	-3.489	355 $[M-H-CO_2]^-$, 330 $[M-H-prenyl]^-$, 287, 275, 262, 219
Pyrone derivative 6	$C_{25}H_{31}O_8$	neg	16.08	10.5	459.201	459.2024	-3.16	305, 293, 153 $[C_8H_9O_3]^-$
unknown prenylated	$C_{26}H_{37}O_5$	neg	16.21	8.5	429.2628	429.2646	-4.257	360 $[M-H-prenyl]^-$, 290, 289, 247, 235, 222
Lupulone	$C_{26}H_{37}O_4$	neg	16.41	8.5	413.2677	413.2697	-4.992	369 $[M-H-CO_2]^-$, 344 $[M-H-prenyl]^-$, 301 $[M-H-prenyl-C_3H_7]^-$, 289, 276, 233, 208, 152
Pyrone derivative 8	$C_{24}H_{27}O_7$	neg	16.94	11.5	427.175	427.1762	-2.965	261, 153 $[C_8H_9O_3]^-$
Auricepyron analogue 2	$C_{25}H_{31}O_7$	neg	17.11	10.5	443.206	443.2075	-3.467	399, 289, 277, 261, 193, 153 $[C_8H_9O_3]^-$
unknown	$C_{29}H_{37}O_8$	neg	17.3	11.5	513.2473	513.2494	-4.055	359, 347, 289, 277
3-[[3-(3,7-dimethyl-2,6-octadien-1-yl)-2,4,6-trihydroxy-5-(2-methyl-1-oxobutyl)phenyl]methyl]-4-hydroxy-5,6-dimethyl-2H-pyran-2-one	$C_{29}H_{37}O_7$	neg	17.36	11.5	497.2529	497.2545	-3.111	357, 345, 343, 331
unknown	$C_{30}H_{39}O_7$	neg	17.77	11.5	511.2685	511.2701	-3.26	467 $[M-H-CO_2]^-$, 357, 345, 331

Table S9. Annotated compounds in the methanol extract of *Helichrysum taenari* Rothm. (HT) based on chromatographic and spectrometric data using UPLC-HRMS/MS in negative and positive ESI mode.

Compound	EC $[M-H]^-$ / $[M+H]^+$	IM	Rt (min)	RDBeq	Experimental *m/z*	Theoretical *m/z*	Δm (ppm)	MS/MS Fragments
Quinic acid	$C_7H_{11}O_6$	neg	0.87	2.5	191.0555	191.0561	-3.304	-
Malic acid	$C_4H_5O_5$	neg	0.93	2.5	133.0138	133.0142	-3.658	-
Caffeoylquinic acid 2	$C_{16}H_{17}O_9$	neg	3.66	8.5	353.0866	353.0878	-3.3	191, 179, 161, 135
Caffeoyl hexoside	$C_{15}H_{17}O_9$	neg	4.01	7.5	341.0865	341.0878	-3.68	179 $[M-H-hexosyl]^-$, 135 $[M-H-hexosyl-CO_2]^-$
Caffeoylquinic acid 3	$C_{16}H_{17}O_9$	neg	4.49	8.5	353.0862	353.0878	-4.688	191, 179, 135
Caffeic acid	$C_9H_7O_4$	neg	4.91	6.5	179.0343	179.035	-3.865	161, 135, 109
Caffeoyl glycerol	$C_{12}H_{13}O_6$	neg	5	6.5	253.0708	253.0718	-3.68	-
unknown	$C_{19}H_{19}O_{11}$	neg	5.16	10.5	423.0918	423.0933	-3.509	379 $[M-H-CO_2]^-$, 287, 261, 217
Kaempferol diglycoside	$C_{27}H_{29}O_{16}$	neg	5.25	13.5	609.1438	609.1461	-3.789	-
5-*O*-Feruloylquinic acid	$C_{17}H_{19}O_9$	neg	5.37	8.5	367.1024	367.1035	-2.82	193, 191, 173, 134
Quercetin glycoside 2	$C_{21}H_{19}O_{12}$	neg	5.82	12.5	463.0862	463.0882	-4.295	301, 179

Dicaffeoylquinic acid 2	$C_{25}H_{23}O_{12}$	neg	6.02	14.5	515.1175	515.1195	-3.901	353, 203, 191, 179, 173
Kaempferol-3-*O*-glucoside	$C_{21}H_{19}O_{11}$	neg	6.13	12.5	447.0918	447.0933	-3.388	327, 285, 284, 283, 255, 227, 211, 181, 175
Dicaffeoylquinic acid 3	$C_{25}H_{23}O_{12}$	neg	6.22	14.5	515.1172	515.1195	-4.367	353, 203, 191, 179, 173, 161
Apigenin-6-*C*-glucoside	$C_{21}H_{19}O_{10}$	neg	6.3	12.5	431.0971	431.0984	-2.992	-
Dicaffeoylquinic acid 4	$C_{25}H_{23}O_{12}$	neg	6.45	14.5	515.118	515.1195	-2.949	353, 203, 191, 179, 173, 161
unknown	$C_{25}H_{25}O_{11}$	neg	6.5	13.5	501.1385	501.1402	-3.422	339, 323, 177
Luteolin glycoside	$C_{21}H_{19}O_{11}$	neg	6.56	12.5	447.0918	447.0933	-3.321	285, 175, 151, 133
unknown	$C_9H_{15}O_4$	neg	6.7	2.5	187.0969	187.0976	-3.54	169 $[M-H-H_2O]^-$, 125 $[M-H-H_2O-CO_2]^-$
Kaempferol dihexoside 1	$C_{30}H_{25}O_{14}$	neg	6.81	18.5	609.1223	609.125	-4.479	447, 285
Kaempferol or luteolin derivative	$C_{29}H_{27}O_{14}$	neg	7.14	16.5	599.1385	599.1406	-3.569	459, 447
Tiliroside	$C_{30}H_{25}O_{13}$	neg	7.27	18.5	593.1279	593.1301	-3.699	447, 307, 285
Tribuloside	$C_{30}H_{25}O_{13}$	neg	7.44	18.5	593.1282	593.1301	-3.193	447, 307, 285
Luteolin	$C_{15}H_9O_6$	neg	7.52	11.5	285.0396	285.0405	-3.197	285, 199, 175, 151, 133, 107
unknown	$C_{22}H_{17}O_8$	neg	7.7	14.5	409.0915	409.0929	-3.424	391 $[M-H-H_2O]^-$
Apigenin	$C_{15}H_9O_5$	neg	8.15	11.5	269.0443	269.0455	-4.82	225, 201, 151
unknown	$C_{17}H_{21}O_7$	neg	8.56	7.5	337.1281	337.1293	-3.37	293 $[M-H-CO_2]^-$
C18 Trihydroxy fatty acid 2	$C_{18}H_{33}O_5$	neg	8.69	2.5	329.2321	329.2333	-3.91	311, 309 $[M-H_2O]^-$, 293, 291 $[M-2H_2O]^-$, 229, 211, 183, 171
Flavonoid derivative	$C_{23}H_{17}O_8$	neg	8.97	15.5	421.0915	421.0929	-3.255	281, 269
unknown	$C_{18}H_{21}O_7$	neg	9.22	8.5	349.128	349.1293	-3.684	331 $[M-H-H_2O]^-$, 277, 249
unknown	$C_{22}H_{27}O_9$	neg	9.38	9.5	435.1648	436.1661	-2.816	-
Arzanol analogue	$C_{22}H_{27}O_8$	neg	9.52	9.5	419.1696	419.1711	-3.7	401, 319
Terpene 1	$C_{17}H_{25}O_4$	neg	10.44	5.5	293.1749	293.1758	-3.181	236, 221
Methylated flavonoid 17	$C_{18}H_{17}O_7$	pos	12.37	10.5	345.0965	345.0969	-1.012	300 $[M+H-CH_3]^+$, 315 $[M+H-2CH_3]^+$
Pinostrobin	$C_{16}H_{15}O_4$	pos	12.67	9.5	271.0965	271.0965	-0.057	167, 131
C17 Hydroxy fatty acid 1	$C_{17}H_{36}O_5$	pos	12.93	0	320.2558	320.2557	0.326	302 $[M+H-H_2O]^-$
unknown	$C_{22}H_{25}O_6$	pos	13.04	10.5	385.1646	385.1646	-0.039	367 $[M+H-H_2O]^+$, 315
Triterpene 3	$C_{29}H_{47}O$	pos	13.22	6.5	411.362	411.3621	-0.347	393, 327, 309, 295, 271, 255, 253, 215
unknown	$C_{22}H_{25}O_6$	pos	13.28	10.5	385.1643	385.1646	-0.662	367 $[M+H-H_2O]^+$, 315
C17 Hydroxy fatty acid 2	$C_{17}H_{34}O_5$	pos	13.37	1	318.2401	318.2401	0.014	300 $[M+H-H_2O]^+$

Table S10. Annotated compounds in the methanol extract of *Helichrysum italicum* (Roth) G.Don subsp. *italicum* (HII) based on chromatographic and spectrometric data using UPLC-HRMS/MS in negative and positive ESI mode.

Compound	EC $[M-H]^-$ / $[M+H]^+$	IM	Rt (min)	RDBeq	Experimental *m/z*	Theoretical *m/z*	Δm (ppm)	MS/MS Fragments
Quinic acid	$C_7H_{11}O_6$	neg	0.84	2.5	191.0554	191.0561	-3.88	173, 171, 127
Malic acid	$C_4H_5O_5$	neg	0.94	2.5	133.0137	133.0142	-4.41	115
Protocatechuic acid -*O*-hexoside	$C_{13}H_{15}O_9$	neg	2.21	6.5	315.0711	315.0722	-3.318	153, 152, 109, 108
Caffeoylquinic acid 1	$C_{16}H_{17}O_9$	neg	2.6	8.5	353.0865	353.0878	-3.555	191, 179, 173, 161, 135
2,4-Dihydroxybenzoic acid	$C_7H_5O_4$	neg	2.94	5.5	153.0188	153.0193	-3.672	109
Caffeoylquinic acid 2	$C_{16}H_{17}O_9$	neg	3.48	8.5	353.0865	353.0878	-3.64	191, 179, 173, 161, 135
Aesculin	$C_{15}H_{15}O_9$	neg	4.05	8.5	339.071	339.0722	-3.466	177
unknown	$C_{21}H_{17}O_{13}$	neg	4.27	13.5	477.0659	477.0675	-3.362	-
Caffeoyl hexoside	$C_{15}H_{17}O_9$	neg	4.36	7.5	341.0865	341.0878	-3.944	179, 135
Caffeoylquinic acid 3	$C_{16}H_{17}O_9$	neg	4.43	8.5	353.0862	353.0878	-4.688	191, 179, 173, 161, 135
Caffeic acid	$C_9H_7O_4$	neg	4.85	6.5	179.0344	179.035	-3.363	161, 135
Dicaffeoylquinic acid 1	$C_{25}H_{23}O_{12}$	neg	5.06	14.5	515.1176	515.1195	-3.668	353, 203, 191, 179, 173, 161
Myricetin-3-*O*-glucoside	$C_{21}H_{19}O_{13}$	neg	5.22	12.5	479.0814	479.0831	-3.661	317
Delphinidin-3-glycoside	$C_{21}H_{21}O_{12}$	neg	5.56	11.5	465.102	465.1038	-3.933	303
Myricetin malonylhexoside	$C_{24}H_{21}O_{16}$	neg	5.7	14.5	565.0813	565.0835	-3.907	521, 317
Quercetin glycoside 2	$C_{21}H_{19}O_{12}$	neg	5.78	12.5	463.0861	463.0882	-4.49	301, 300, 179, 151

Dicaffeoylquinic acid 2	$C_{25}H_{23}O_{12}$	neg	6.11	14.5	515.117	515.1195	-4.852	353, 203, 191, 179, 173, 161
Malonyl-dicaffeoylquinic acid	$C_{28}H_{25}O_{15}$	neg	6.22	16.5	601.1172	601.1199	-4.497	557, 515, 439, 395, 377
Eriodictyol-*O*-hexoside	$C_{21}H_{21}O_{11}$	neg	6.44	11.5	449.107	449.1089	-4.33	287, 151, 135
Quercetin glycoside 3	$C_{21}H_{19}O_{12}$	neg	6.48	12.5	463.0864	463.0882	-3.885	301, 179, 151
unknown	$C_9H_{15}O_4$	neg	6.67	2.5	187.0968	187.0976	-4.234	169 $[M-H-H_2O]^-$, 125 $[M-H-H_2O-CO_2]^-$
4-Feruloyl-5-caffeoylquinic acid	$C_{26}H_{25}O_{12}$	neg	6.77	14.5	529.1334	529.1351	-3.382	367, 353, 193
Kaempferol dihexoside 1	$C_{30}H_{25}O_{14}$	neg	6.84	18.5	609.1223	609.125	-4.381	463, 447, 323, 285, 241, 229
Quercetin	$C_{15}H_9O_7$	neg	7.44	11.5	301.0342	301.0354	-3.973	178, 151
unknown	$C_{26}H_{29}O_{13}$	neg	7.51	12.5	549.1592	549.1614	-3.868	-
Hydroxycinnamic acid derivative 1	$C_{21}H_{27}O_9$	neg	7.68	8.5	423.1644	423.1661	-3.842	179 $[C_9H_7O_4]^-$, 161 $[C_9H_7O_4-H_2O]^-$
C18 Trihydroxy fatty acid 1	$C_{18}H_{31}O_5$	neg	8.2	3.5	327.2164	327.2177	-4.056	309 $[M-H_2O]^-$, 291 $[M-2H_2O]^-$, 273 $[M-3H_2O]^-$, 239, 229, 211, 183, 171
Hydroxycinnamic acid derivative 2	$C_{25}H_{27}O_{10}$	neg	8.4	12.5	487.1593	487.161	-3.428	179 $[C_9H_7O_4]^-$, 161 $[C_9H_7O_4-H_2O]^-$
C18 Trihydroxy fatty acid 2	$C_{18}H_{33}O_5$	neg	8.64	2.5	329.232	329.2333	-4.001	311 $[M-H_2O]^-$, 293 $[M-2H_2O]^-$, 275 $[M-3H_2O]^-$, 229, 211, 183, 171
unknown	$C_{17}H_{13}O_6$	neg	8.8	11.5	313.0707	313.0718	-3.358	295 $[M-H-H_2O]^-$, 269 $[M-H-CO_2]^-$
Dilignol analogue 2	$C_{20}H_{23}O_7$	neg	8.87	9.5	375.1435	375.1449	-3.829	357 $[M-H-H_2O]^-$, 331 $[M-H-CO_2]^-$
unknown	$C_{23}H_{21}O_7$	neg	9.93	13.5	409.1276	409.1293	-4.122	-
Terpene 1	$C_{17}H_{25}O_4$	neg	10.38	5.5	293.1748	293.1758	-3.522	236, 221
Terpene 2	$C_{17}H_{25}O_3$	pos	10.55	5.5	277.1797	277.1809	-0.257	235, 221
C17 hydroxy fatty acid 2	$C_{17}H_{34}O_5$	pos	12.89	1	318.24	318.2412	-0.363	300 $[M+H-H_2O]^+$
unknown	$C_8H_5O_3$	pos	13.9	6.5	149.0235	149.0234	0.869	121 $[M+H-CO]^+$
Triterpenic acid 1	$C_{30}H_{45}O_2$	pos	15.16	8.5	437.3411	437.3425	-0.725	395 $[M+H-CO_2]^+$
Triterpenic acid 2	$C_{30}H_{47}O_2$	pos	15.57	7.5	439.357	439.3582	-0.062	395 $[M+H-CO_2]^+$

Table S11. Annotated compounds in the methanol extract of *Helichrysum italicum* (Roth) G.Don subsp. *microphyllum* (Willd.) Nyman (HIM) based on chromatographic and spectrometric data using UPLC-HRMS/MS in negative ESI mode.

Compound	EC $[M-H]^-$ / $[M+H]^+$	IM	Rt (min)	RDBeq	Experimental *m/z*	Theoretical *m/z*	Δm (ppm)	MS/MS Fragments
Quinic acid	$C_7H_{11}O_6$	neg	0.86	2.5	191.0554	191.0561	-3.723	173, 171, 155, 127, 111, 109
Malic acid	$C_4H_5O_5$	neg	0.97	2.5	133.0137	133.0142	-3.733	115
Protocatechuic acid -*O*-hexoside	$C_{13}H_{15}O_9$	neg	2.3	6.5	315.0711	315.0722	-3.222	153, 109
2-Isopropyl malic acid	$C_7H_{11}O_5$	neg	3.47	2.5	175.0607	175.01612	-2.952	157, 113, 85
Caffeoylquinic acid 2	$C_{16}H_{17}O_9$	neg	3.6	8.5	353.0867	353.0878	-3.215	191, 179, 161, 135
Hydroxycinnamic acid derivative	$C_{15}H_{19}O_9$	neg	4	6.5	343.1023	343.1035	-3.484	297, 179, 135
7-*O*-*β*-(*D*-glucopyranosyl)-5-methoxy-1(3H)-isobenzofuranone or 6-*O*-(*β*-*D*-glucopyranosyloxy)-4-methoxy-1(3H)-isobenzofuranone	$C_{15}H_{17}O_9$	neg	4.38	7.5	341.0867	341.0878	-3.153	179 $[M-H-hexosyl]^-$, 135 $[M-H-hexosyl-CO_2]^-$
Caffeoylquinic acid 3	$C_{16}H_{17}O_9$	neg	4.48	8.5	353.0862	353.0878	-4.518	191, 179, 135, 111
Caffeic acid	$C_9H_7O_4$	neg	4.9	6.5	179.0343	179.035	-3.977	161, 135, 107
Gnaphaliol-*O*-*β*-*D*-glucopyranoside as formate adduct 441 [C20H25O11]-	$C_{20}H_{25}O_{11}$	neg	5.35	8.5	395.1336	395.1348	-3.026	395 $[M-H-COOH]^-$, 215 $[395-hexose]^-$
Quercetin glycoside 1	$C_{21}H_{19}O_{12}$	neg	5.4	12.5	463.0862	463.0882	-4.295	301
unknown	$C_{20}H_{27}O_{10}$	neg	5.7	7.5	427.1596	427.161	-3.255	381, 363, 219
Hydroxyjasmonate	$C_{12}H_{17}O_4$	neg	5.75	4.5	225.1125	225.1132	-3.12	207, 181, 151
Quercetin glycoside 2	$C_{21}H_{19}O_{12}$	neg	5.8	12.5	463.0862	463.0882	-4.231	301
unknown	$C_{25}H_{33}O_{14}$	neg	5.96	9.5	557.1855	557.1876	-3.749	511
Dicaffeoylquinic acid 3	$C_{25}H_{23}O_{12}$	neg	6.18	14.5	515.1172	515.1195	-4.483	353, 317, 299, 255, 203
Quercetin glycoside 3	$C_{21}H_{19}O_{12}$	neg	6.56	12.5	463.0868	463.0882	-3.108	301
unknown	$C_{13}H_{19}O_4$	neg	6.78	4.5	239.1281	239.1289	-3.481	195 $[M-H-CO_2]^-$, 137, 101, 99

unknown	$C_{21}H_{27}O_9$	neg	7.2	8.5	423.1646	423.1661	-3.416	405 $[M-H-H_2O]^-$, 335, 293, 275, 243, 201, 167, 137
Tiliroside	$C_{30}H_{25}O_{13}$	neg	7.3	18.5	593.1279	593.1301	-3.598	447, 307, 285
Quercetin coumaroylglucoside analogue	$C_{30}H_{29}O_{15}$	neg	7.36	16.5	629.149	629.1512	-3.502	463, 301
unknown	$C_{15}H_{15}O_7$	neg	7.39	8.5	307.0814	307.0823	-2.951	195
Luteolin	$C_{15}H_9O_6$	neg	7.42	11.5	285.0396	285.0405	-3.092	257, 241, 223, 217, 213, 199, 197, 175
7-(2,3-dihydroxy-3-methylbutoxy)-5-hydroxy-6-methoxy-2H-1-benzopyran-2-one	$C_{15}H_{17}O_7$	neg	7.5	7.5	309.0968	309.098	-3.902	291 $[M-H-H_2O]^-$, 265 $[M-H-CO_2]^-$, 247, 237, 196 $[M-H-CO_2\text{-prenyl}]^-$
Quercetin	$C_{15}H_9O_7$	neg	7.51	11.5	301.0345	301.0354	-3.076	178, 151
unknown	$C_{12}H_{13}O_5$	neg	7.54	6.5	237.0759	237.0768	-3.825	219 $[M-H-H_2O]^-$, 193 $[M-H-CO_2]^-$, 149
unknown	$C_{16}H_{19}O_6$	neg	7.6	7.5	307.1176	307.1187	-3.782	289 $[M-H-H_2O]^-$, 195, 167
Isorhamnetin	$C_{16}H_{11}O_7$	neg	7.78	11.5	315.0497	315.051	-4.177	300, 283, 271, 255, 243
unknown	$C_{16}H_9O_6$	neg	7.82	12.5	297.0395	297.0405	-3.169	269 $[M-H-CO]^-$
6-ethyl-4-hydroxy-5-methyl-3-(3-oxopentyl)-2H-pyran-2-one	$C_{13}H_{17}O_4$	neg	7.9	5.5	237.1123	237.1132	-4.058	193 $[M-H-CO_2]^-$
Naringenin	$C_{15}H_{11}O_5$	neg	8.2	10.5	271.0602	271.0612	-3.53	177, 151, 107, 65
unknown	$C_{21}H_{23}O_8$	neg	8.26	10.5	403.1383	403.1398	-3.872	385 $[M-H-H_2O]^-$, 317, 289, 249
Homoeriodictyol	$C_{16}H_{13}O_6$	neg	8.31	10.5	301.0706	301.0718	-3.791	286, 273, 242, 177, 165, 151, 107
unknown	$C_{21}H_{25}O_8$	neg	8.38	9.5	405.1541	405.1555	-3.408	387 $[M-H-H_2O]^-$, 319, 291, 251
a) 3,5-dimethyl-6-isopropyl-4-methoxy-a-pyron, b) 2-methoxy-3,5-dimethyl-6-(1-methylethyl)-4H-pyran-4-one	$C_{11}H_{15}O_3$	neg	8.43	4.5	195.1019	195.1027	-3.73	151 $[M-H-CO_2]^-$
Pinobanskin	$C_{15}H_{11}O_5$	neg	8.5	10.5	271.0601	271.0612	-3.972	253, 225, 215, 209, 197, 151
Coumarin analogue	$C_{15}H_{17}O_7$	neg	8.67	7.5	309.0967	309.098	-3.999	265 $[M-H-CO_2]^-$, 247 $[M-H-CO_2-H_2O]^-$
Micropyrone	$C_{14}H_{19}O_4$	neg	8.76	5.5	251.1279	251.1289	-3.991	207 $[M-H-CO_2]^-$
unknown	$C_{21}H_{25}O_8$	neg	8.94	9.5	405.1541	405.1555	-3.408	387 $[M-H-H_2O]^-$, 336
unknown	$C_{19}H_{19}O_9$	neg	9.05	10.5	391.1019	391.1035	-3.977	237, 225, 181
Trihydroxy-methoxyflavone	$C_{16}H_{11}O_6$	neg	9.11	11.5	299.0552	299.0561	-3.114	284, 256, 239, 211
unknown	$C_{16}H_{17}O_7$	neg	9.19	8.5	321.0967	321.098	-3.85	277 $[M-H-CO_2]^-$, 249
Araneophthalide	$C_{15}H_{17}O_6$	neg	9.41	7.5	293.108	293.1031	-4.27	249 $[M-H-CO_2]^-$
unknown	$C_{17}H_{19}O_8$	neg	9.78	8.5	351.1074	351.1085	-3.278	319, 291, 275, 261, 235
unknown	$C_{23}H_{21}O_7$	neg	9.95	13.5	409.1279	409.1293	-3.437	391 $[M-H-H_2O]^-$, 381, 323, 295
Pinocembrin	$C_{15}H_{11}O_4$	neg	10.16	10.5	255.0653	255.0663	-4.007	213, 187, 185, 169, 151, 145, 107
6-Demethylacronylin	$C_{13}H_{15}O_4$	neg	10.34	6.5	235.0966	235.0976	-4.178	191 $[C_{12}H_{15}O_2]^-$
5,7-Dihydroxy-3-methoxyflavone	$C_{16}H_{11}O_5$	neg	10.57	11.5	283.0603	283.0612	-3.062	268, 239, 211
Plicatipyrone	$C_{22}H_{25}O_8$	neg	11.06	10.5	417.1542	417.1555	-2.495	251, 153
unknown	$C_{21}H_{25}O_7$	neg	11.17	9.5	389.1596	389.1606	-2.483	371 $[M-H-H_2O]^-$, 317, 303, 277, 263, 251
Plicatipyrone analogue 1	$C_{22}H_{25}O_8$	neg	11.87	10.5	417.1542	417.1555	-2.999	251, 153
Arenol	$C_{21}H_{23}O_7$	neg	12.01	10.5	387.144	387.1449	-2.522	275, 247, 235, 139
unknown	$C_{21}H_{25}O_6$	neg	12.08	9.5	373.1645	373.1657	-3.22	287, 261, 247, 235
Arzanol	$C_{22}H_{25}O_7$	neg	12.86	10.5	401.1586	401.1606	-4.927	247, 235, 205, 191, 166, 153
Plicatipyrone analogue 2	$C_{22}H_{25}O_8$	neg	13.32	10.5	417.1539	417.1555	-3.742	263, 251
3-Methylarzanol	$C_{23}H_{27}O_7$	neg	13.67	10.5	415.175	415.1762	-3.05	261, 249, 247, 235, 205, 193, 180, 153
Heterodimer pyrone-phloroglucinol	$C_{23}H_{27}O_8$	neg	14.05	10.5	431.1696	431.1711	-3.667	263, 251
6-*O*-desmethylauricepyron or arenol C analogue	$C_{24}H_{29}O_7$	neg	14.25	10.5	429.1906	429.1919	-2.951	275, 263, 247, 235, 153

Heliarzanol analogue	$C_{24}H_{29}O_8$	neg	15.01	10.5	445.1853	445.1868	-3.417	291, 279, 261, 193
Auricepyron analogue	$C_{25}H_{31}O_7$	neg	15.14	10.5	443.2059	443.2075	-3.738	289, 277, 263, 249, 235, 153
Pyrone derivative 4	$C_{25}H_{31}O_8$	neg	15.64	10.5	459.2007	459.2024	-3.835	305, 293, 291, 279
Italipyrone	$C_{22}H_{23}O_7$	neg	15.73	11.5	399.1436	399.1449	-3.198	245, 233, 153
unknown	$C_{26}H_{27}O_{10}$	neg	16	13.5	499.1588	499.161	-4.268	333
unknown	$C_{26}H_{33}O_7$	neg	16.16	10.5	457.2219	457.2232	-2.814	289, 277, 275, 263
unknown	$C_{26}H_{33}O_8$	neg	16.35	10.5	473.2166	473.2181	-3.257	305, 293, 291, 279
unknown	$C_{27}H_{29}O_{10}$	neg	16.7	13.5	513.1746	513.1766	-3.898	347, 333
unknown	$C_{27}H_{31}O_9$	neg	16.91	12.5	499.1957	499.1974	-3.297	430, 333

Table S12. Annotated compounds in the methanol extract of *Helichrysum stoechas* (L.) Moench subsp. *barrelieri* (Ten.) Nyman (HSB) based on chromatographic and spectrometric data using UPLC-HRMS/MS in negative and positive ESI mode.

Compound	EC $[M-H]^-$ / $[M+H]^+$	IM	Rt (min)	RDBeq	Experimental *m/z*	Theoretical *m/z*	Δm (ppm)	MS/MS Fragments
Quinic acid	$C_7H_{11}O_6$	neg	0.84	2.5	191.0555	191.0561	-3.304	173, 171, 155, 127, 111, 109
Malic acid	$C_4H_5O_5$	neg	0.94	2.5	133.0137	133.0142	-3.959	115
Protocatechuic acid-*O*-hexoside	$C_{13}H_{15}O_9$	neg	2.25	6.5	315.0713	315.0722	-2.841	153, 152, 109, 108
Caffeoylquinic acid 1	$C_{16}H_{17}O_9$	neg	2.6	8.5	353.0869	353.0878	-2.621	191, 179, 173, 161, 135
Protocatechuic acid	$C_7H_5O_4$	neg	2.99	5.5	153.0189	153.0193	-2.758	109, 95, 79, 59
Caffeoyl hexoside	$C_{15}H_{17}O_9$	neg	4.37	7.5	341.0865	341.0878	-3.856	179, 135
Caffeoylquinic acid 3	$C_{16}H_{17}O_9$	neg	4.45	8.5	353.0861	353.0878	-4.858	191, 179, 173, 161, 135
Caffeic acid	$C_9H_7O_4$	neg	4.88	6.5	179.0344	179.035	-3.474	161, 135, 121
Dicaffeoylquinic acid isomer 1	$C_{25}H_{23}O_{12}$	neg	5.05	14.5	515.1175	515.1195	-3.901	353, 203, 191, 179, 173, 161, 135
Myricetin-3-*O*-glucoside	$C_{21}H_{19}O_{13}$	neg	5.31	12.5	479.0818	479.0831	-2.701	317
5-*O*-Feruloylquinic acid	$C_{17}H_{19}O_9$	neg	5.39	8.5	367.1023	367.1035	-3.175	193, 191, 173, 134
unknown	$C_{23}H_{21}O_{10}$	neg	5.41	13.5	457.1158	457.114	3.85	-
Quercetin glycoside 2	$C_{21}H_{19}O_{12}$	neg	5.78	12.5	463.0863	463.0882	-4.101	301, 300, 273, 255, 179, 151
Dicaffeoylquinic acid isomer 2	$C_{25}H_{23}O_{12}$	neg	5.96	14.5	515.1175	515.1195	-3.901	353, 203, 191, 179, 173, 161
Malonyl-dicaffeoylquinic acid	$C_{28}H_{25}O_{15}$	neg	6.22	16.5	601.1173	601.1199	-4.297	557, 515, 439, 395, 377
unknown	$C_{23}H_{19}O_9$	neg	6.42	14.5	439.1053	439.1035	4.246	-
unknown	$C_{25}H_{25}O_{11}$	neg	6.47	13.5	501.1386	501.1402	-3.302	-
Quercetin glycoside 3	$C_{21}H_{19}O_{12}$	neg	6.49	12.5	463.0867	463.0882	-3.237	301, 273, 179, 151
unknown	$C_9H_{15}O_4$	neg	6.67	2.5	187.0969	187.0976	-3.593	169 $[M-H-H_2O]^-$, 125 $[M-H-H_2O-CO_2]^-$
Feruloyl-caffeoyl-quinic acid	$C_{26}H_{25}O_{12}$	neg	6.79	14.5	529.1334	529.1351	-3.268	367, 353, 191
Kaempferol dihexoside 1	$C_{30}H_{25}O_{14}$	neg	6.84	18.5	609.1222	609.125	-4.578	463, 447, 323, 285, 241, 229
Quercetin-7-*O*-(caffeoyl)-hexoside	$C_{30}H_{25}O_{15}$	neg	7.08	18.5	625.1172	625.1199	-4.324	463, 445, 323, 301, 273, 257, 179
Tiliroside	$C_{30}H_{25}O_{13}$	neg	7.24	18.5	593.1275	593.1301	-4.323	447, 307, 285, 257, 241, 229, 213
Quercetin	$C_{15}H_9O_7$	neg	7.46	11.5	301.0342	301.0354	-3.973	283, 271, 257, 179, 151, 107
unknown	$C_{23}H_{25}O_9$	neg	7.54	11.5	445.1487	445.1504	-3.876	-
Hydroxycinnamic acid derivative 1	$C_{21}H_{27}O_9$	neg	7.7	8.5	423.1644	423.1661	-3.912	179 $[C_9H_7O_4]^-$, 161 $[C_9H_7O_4-H_2O]^-$
C18 Trihydroxy fatty acid 1	$C_{18}H_{31}O_5$	neg	8.16	3.5	327.2164	327.2177	-3.873	309 $[M-H_2O]^-$, 291 $[M-2H_2O]^-$, 273 $[M-3H_2O]^-$, 239, 229, 211, 183, 171

Hydroxycinnamic acid derivative 2	$C_{25}H_{27}O_{10}$	neg	8.4	12.5	487.1593	487.161	-3.49	179 $[C_9H_7O_4]^-$, 161 $[C_9H_7O_4\text{-}H_2O]^-$
C18 Trihydroxy fatty acid 2	$C_{18}H_{33}O_5$	neg	8.56	2.5	329.2321	329.2333	-3.637	311 $[M\text{-}H_2O]^-$, 293 $[M\text{-}2H_2O]^-$, 275 $[M\text{-}3H_2O]^-$, 229, 211, 183, 171
unknown	$C_{17}H_{13}O_6$	neg	8.79	11.5	313.0707	313.0718	-3.454	295 $[M\text{-}H\text{-}H_2O]^-$, 269 $[M\text{-}H\text{-}CO_2]^-$
Dilignol analogue 2	$C_{20}H_{23}O_7$	neg	8.85	9.5	375.1437	375.1449	-3.162	357 $[M\text{-}H\text{-}H_2O]^-$, 331 $[M\text{-}H\text{-}CO_2]^-$
unknown	$C_{22}H_{19}O_7$	neg	9.48	13.5	395.112	395.1136	-4.116	377 $[M\text{-}H\text{-}H_2O]^-$
Pinocembrin	$C_{15}H_{11}O_4$	neg	10.14	10.5	255.0652	255.0663	-4.243	-
Terpene 1	$C_{17}H_{25}O_4$	neg	10.4	5.5	293.175	293.1758	-2.772	236, 221
Terpene 2	$C_{17}H_{25}O_3$	pos	10.55	5.5	277.1797	277.1798	-0.257	235, 221
Methylated flavonoid 15	$C_{18}H_{17}O_7$	pos	12	10.5	345.0967	345.0969	-0.404	330 $[M+H\text{-}CH_3]^+$, 315 $[M+H\text{-}2CH_3]^+$
C17 Hydroxy fatty acid 2	$C_{17}H_{34}O_5$	pos	12.92	1	318.2399	318.2401	-0.458	300 $[M+H\text{-}H_2O]^+$
unknown	$C_8H_5O_3$	pos	13.92	6.5	149.0233	149.0233	-0.272	-
unknown	$C_{23}H_{33}O_3$	pos	15.01	7.5	357.2421	357.2424	-0.9	-
Triterpenic acid 1	$C_{30}H_{45}O_2$	pos	15.25	8.5	437.341	437.3414	-1	395 $[M+H\text{-}CO_2]^+$
unknown	$C_{21}H_{42}O_5$	pos	15.34	1	374.3025	374.3027	-0.416	-
Triterpenic acid 2	$C_{30}H_{47}O_2$	pos	15.6	7.5	439.357	439.3571	-0.221	395 $[M+H\text{-}CO_2]^+$

References

1. Dimopoulos, P.; Raus, T.; Bergmeier, E.; Constantinidis, T.; latrou, G.; Kokkini, S.; Tzanoudakis, D. *Vascular Plants of Greece: An Annotated Checklist*, 1st ed.; Botanischer Garten und Botanisches Museum Berlin-Dahlem & Hellenic Botanical Society: Athens, Greece, 2013; As regularly updated in Flora of Greece Web, available online: https://portal.cybertaxonomy.org/flora-greece/intro (accessed on 12 December 2024).
2. Kougioumoutzis, K.; Kokkoris, I.P.; Panitsa, M.; Strid, A.; Dimopoulos, P. Extinction Risk Assessment of the Greek Endemic Flora. *Biology* **2021**, *10*, 195, https://doi.org/10.3390/biology10030195.
